# Importance of balanced datasets with feature selection and ensemble methods on heart disease classification using distinctive machine learning techniques: a comparative analysis

**DOI:** 10.1038/s41598-026-47691-4

**Published:** 2026-04-07

**Authors:** Jinat Ara, Hanif Bhuiyan, Isfara Islam Roza, Abrar Shadman Mohammad Nahin

**Affiliations:** 1 Department of Electrical Engineering and Information Systems, University of Pannonia, Veszprem, Hungary; 2 Data Scientist, Performance & Analytics Group, City of Gold Coast, Bundall, QLD 4217 Australia; 3 Department of Computer Science and Engineering, Ahsanullah University of Science and Technology, Dhaka, Bangladesh

**Keywords:** Heart disease classification and prediction, Machine Learning models, Data balancing, Feature importance, Ensemble technique, Cardiology, Computational biology and bioinformatics, Diseases, Health care, Mathematics and computing, Medical research, Risk factors

## Abstract

Heart disease, a leading cause of death worldwide, accounts for 31% of global fatalities and requires effective early detection methods to combat its rising prevalence. Early detection and prediction of heart disease remain one of the most pressing challenges in current healthcare. In recent years, machine learning (ML) technologies have offered opportunities to address these inequities by improving heart disease detection and prediction capabilities. This study offers a comparative evaluation of seven machine learning models: Logistic Regression (LR), Decision Tree (DT), Random Forest (RF), Naïve Bayes (NB), Support Vector Machine (SVM), Artificial Neural Networks (ANN), and K-Nearest Neighbors (KNN) for classifying heart disease. Using the ‘BRFSS 2020 Heart Disease Dataset’, this research examines the effects of dataset balancing with various feature selection techniques and an ensemble method with bagging on classification and prediction accuracy. Three feature selection methods ANOVA, Chi-Square, and Regression Analysis were tested through eight different combinations based on union and intersection of these methods: (i) {ANOVA ∪ Chi-Square}, (ii) {ANOVA ∪ Regression}, (iii) {Chi-Square ∪ Regression}, (iv) {ANOVA ∪ Chi-Square ∪ Regression}, (v) {ANOVA ∩ Chi-Square}, (vi) {ANOVA ∩ Regression}, (vii) {Chi-Square ∩ Regression}, and (viii) {ANOVA ∩ Chi-Square ∩ Regression}. Experimental results demonstrate that with a balanced dataset, RF and DT achieved the highest accuracies of 85% and 82%, respectively. Besides, the outcome of the balanced dataset incorporating feature selection techniques indicates that ANOVA-based feature selection was associated with higher performance under the {ANOVA ∪ Chi-Square} and {ANOVA ∪ Chi-Square ∪ Regression} feature combinations, where RF reached the highest accuracy (92%), recall (93%), and AUC score (0.92). Additionally, bagging-based ensemble techniques improved performance for certain high-variance models (DT, RF, and ANN) when applied to the balanced dataset, although the impact varied across models. Despite promising accuracy with dataset balancing incorporating an ensemble method, the recall and AUC scores were relatively low, indicating many positive cases were missing. Consequently, dataset balancing combined with feature selection techniques showed comparatively improved performance across several evaluation metrics under the specific experimental setup. These findings provide comparative insights into preprocessing strategies and optimal machine learning models for heart disease classification, which would be helpful for future research.

## Introduction

Heart disease remains one of the leading causes of death worldwide, a reality that underscores the importance of early detection. Heart disease encompasses a variety of conditions, such as heart attacks, cardiac failure, arrhythmias, and blockages in blood vessels^1–4^. In 2016, heart disease was the cause of roughly 17.9 million deaths, which accounted for 31% of all global deaths, according to the World Health Organization^3,5^. 7.4 million of these deaths were attributable to coronary heart disease, while 6.7 million were caused by stroke^6^. Therefore, there is an urgent need for timely diagnosis and treatment due to the substantial number of individuals who are presently afflicted with heart disease. Without these, the death toll is projected to rise to around 22 million by 2030^7,8^.

Nowadays, accurate identification of heart disease has become increasingly critical in modern healthcare, although human limitations can contribute to delays in diagnosis, which may result in severe consequences. Besides, traditional diagnostic methods are often costly, computationally complex, and time-consuming compared to automated approaches^9,10^. As a result, there is a growing need for sophisticated methods to facilitate early detection of cardiac disease.

Among several approaches, automated heart disease detection provides a practical solution to these challenges. These methods allow us to apply advanced data processing techniques to uncover hidden patterns in medical data, supporting both diagnosis and treatment. Over recent decades, significant progress has been made in various medical fields, including the classification and prediction of heart disease in different conditions^11–14^. In this context, current literature highlights that Machine Learning (ML) can contribute to the medical industry by identifying different patterns in medical data, which is promising for heart disease classification and prediction. According to modern research, integrating ML techniques could lead to the development of improved models capable of supporting the classification and prediction of cardiac disease across diverse scenarios. As ML continues to advance, it has the potential to support cardiac disease diagnosis, improve patient outcomes, and ease the burden on the healthcare system.

In recent years, various studies have used different ML models to classify and predict heart disease. For example, Dun et al.^15^ employed deep learning techniques, considering Random Forests (RF), Logistic Regression (LR), and Support Vector Machines (SVM) to classify heart disease. They incorporated hyperparameter tuning and feature selection techniques and found that neural networks achieved an accuracy of around 78.3%. Similarly, Asl et al.^16^ used SVM to classify heart disease, emphasizing feature selection to improve computational performance. Yaghouby et al.^17^ followed a similar approach, using neural network models such as the Multilayer Perceptron (MLP) classifier, with a focus on feature selection techniques. More details about the existing literature are described in Sect. 2.

Despite these advancements, several challenges need to be addressed for ML predictive models to reach their full potential. One critical issue is ensuring balanced datasets and the quality of data and features used for training and testing. Researchers suggested that the performance of ML models can be significantly enhanced by using balanced datasets^18^. Additionally, the predictive capabilities of these models rely heavily on selecting the right features from the data. Therefore, data balancing and careful feature selection are essential for optimizing model performance and improving classification and prediction accuracy. Furthermore, model performance can also be boosted with ensemble techniques such as bagging.

While many existing models demonstrate their promising ability to improve prediction and classification accuracy, shortcomings in data preprocessing can lead to issues with data standardization, ultimately affecting the model’s effectiveness^19,20^. For example, feature extraction and selection are often overlooked in many models, yet they can be crucial for enhancing classification performance. Furthermore, some researchers suggest that a hybrid algorithmic evaluation process incorporating multiple ML models or algorithms is more effective for improving classification performance than relying on a single optimal approach^21,22^.

However, this work aims to develop a comparative heart disease classification model that incorporates multiple machine learning approaches, modern data processing techniques, and advanced feature selection methods. The aim is to address challenges related to data imbalance and feature selection for enhancing classification and prediction performance. To achieve this, we used seven classification models employing different machine learning algorithms, including (i) Logistic Regression, (ii) Decision Tree (DT), (iii) Random Forest (RF), (iv) Naïve Bayes (NB), (v) Support Vector Machine (SVM), (vi) Artificial Neural Networks (ANN), and (vii) K-Nearest Neighbors (KNN). These models were evaluated using various performance metrics to assess their usefulness. To balance the data, we applied the Synthetic Minority Oversampling Technique (SMOTE) to mitigate class imbalance and improve model performance. For feature selection, we employed three advanced methods {ANOVA, Chi-Square, and Regression} analysis with eight different combinations: (i) {ANOVA ∪ Chi-Square}, (ii) {ANOVA ∪ Regression}, (iii) {Chi-Square ∪ Regression}, (iv) {ANOVA ∪ Chi-Square ∪ Regression}, (v) {ANOVA ∩ Chi-Square}, (vi) {ANOVA ∩ Regression}, (vii) {Chi-Square ∩ Regression}, and (viii) {ANOVA ∩ Chi-Square ∩ Regression}. Additionally, ensemble learning techniques, such as Bagging, were explored as an alternative strategy, and their results were compared with feature selection methods to evaluate their efficiency and effectiveness. The performance of each model was assessed using key metrics (accuracy, precision, recall (from the confusion matrix), and the Receiver Operating Characteristic (ROC) curve) to provide a comprehensive evaluation of its prediction capabilities.

To achieve our goal, we conducted a comprehensive experimental study using real-world heart disease datasets employing multiple machine learning classification models. The dataset used in this research is the BRFSS 2020 Heart Disease Dataset, collected from the Zenodo data repository. It originally included 17 features. The main purpose of our experiments was to answer two key research questions:

- RQ1: What are the impacts of balanced datasets, feature selection, and ensemble techniques on the performance of heart disease classification?
- RQ2: What are the impacts of different Machine Learning (ML) techniques in facilitating heart disease classification?

To validate the performance of each proposed model, we compared its computational accuracy. This comparison enabled us to assess model effectiveness and identify models with comparatively better performance for heart disease classification on the experimental dataset. The results of these comparisons help to address our research questions. The key contributions of this work include:

- Enhanced data reliability through balancing and standardization techniques.
- Employed different feature selection techniques with an advanced combination of feature selection and ensemble methods to observe their relevant impact for improving the performance.
- Employed different Machine Learning (ML) models, such as Logistic Regression (LR), Decision Tree (DT), Random Forest (RF), Naïve Bayes (NB), Support Vector Machine (SVM), Artificial Neural Networks (ANN), and K-Nearest Neighbors (KNN) to facilitate the heart disease classification.
- Distinguished the effect of balanced datasets with feature selection, and ensemble techniques to support and facilitate heart disease classification.
- Distinguished the effect of different Machine Learning (ML) techniques to identify the suitable models for the specific problem.

The remainder of this article is organized as follows: Sect.  2 offers a detailed discussion of the existing literature and its contributing factors. Section  3 provides a detailed description of the proposed model, including the selected data preprocessing techniques, feature selection methods, descriptions of the chosen classifiers, and an in-depth analysis of the experimental results. Section  4 presents a comparative evaluation of the developed ML models with the findings. Finally, Sect.  5 concludes the study with a summary.

## Related literature

According to the researcher’s opinion, machine learning (ML) algorithms are the most suitable technology for the medical industry in disease classification and identification, especially for cardiovascular diseases such as coronary artery disease, heart failure, stroke, and cardiac arrhythmia^23^. Recently, many studies have conducted comparative analyses of various machine learning (ML) algorithms for heart disease classification and prediction. For example, Nagavelli, et al.^24^ provided a brief analysis of different machine learning technologies and their contributions to heart disease detection. Initially, they employed Naïve Bayes (NB) with a weighted approach to predict heart disease. Next, they used a Support Vector Machine (SVM) combined with XGBoost to analyze ischemic heart disease, considering frequency-domain, time-domain, and information-theoretic features. Third, they applied an improved SVM model for heart failure detection using a duality-based optimization scheme. Finally, they proposed an effective heart disease prediction model (HDPM) for a clinical decision support system (CDSS). This system uses outlier detection for noise elimination, the SMOTE-ENN over-sampling technique to balance training data, and XGBoost for prediction. This study demonstrated that ML models are effective and capable of avoiding serious consequences.

Another significant study by Mohan et al.^25^ proposed a hybrid method utilizing two machine learning techniques (hybrid Random Forest with a linear model (HRFLM)) with different combinations of selected features. The proposed HRFLM model improved performance, achieving an accuracy of 88.7%. Following that, Nashif et al.^26^ focused specifically on SVM, which attained 97.53% accuracy, highlighting the effectiveness of Support Vector Machine (SVM) in high-dimensional spaces. Additionally, Biswas et al.^27^ applied Logistic Regression (LR), Support Vector Machine (SVM), Decision Tree (DT), Random Forest (RF), Naïve Bayes (NB), and K-Nearest Neighbor (KNN), using ANOVA, Chi-square, and Mutual Information methods for feature selection, where RF demonstrated superior performance.

Additionally, some comprehensive evaluations have been conducted by Mahmoud et al.^28^ and Apurba et al.^29^. In these two studies, Mahmoud et al.^28^ compared five ML classifiers, including Logistic Regression (LR), Support Vector Classifier (SVC), K-Nearest Neighbors (KNN), Decision Tree (DT), and Random Forest (RF), by experimenting on various datasets. Their comparative evaluation concluded that RF achieved the best accuracy, around 85.05%. Similarly, Apurba et al.^29^ evaluated Naïve Bayes (NB), Decision Tree (DT), Logistic Regression (LR), and Random Forest (RF) models using a large dataset that contained missing values. Their results determined RF’s superiority with 90.16% accuracy. Additionally, in another study, Jan et al.^30^ proposed an ensemble learning method combining five classifiers, including Support Vector Machine (SVM), Artificial Neural Network (ANN), Naïve Bayesian (NB), Regression Analysis (RA), and Random Forest (RF), to predict cardiovascular disease. Their model was implemented on the Cleveland and Hungarian datasets from the UCI repository. The experimental results demonstrated that the ensemble model outperforms others in terms of predictive accuracy. Chowdhury et al.^31^ proposed an ML-based model for heart disease prediction to improve prediction accuracy. They trained their model using several classification algorithms, including Decision Tree (DT), Logistic Regression (LR), K-Nearest Neighbors (KNN), Naive Bayes (NB), and Support Vector Machine (SVM). Their experiments used a custom dataset collected from hospitals and healthcare facilities in the Sylhet region of Bangladesh. The results revealed that SVM showed the best performance, with an accuracy of 91%.

Several researchers have highlighted the importance of data preprocessing and feature selection in enhancing prediction accuracy. For example, Benhar et al.^32^ identified challenges in their study related to data quality, such as inconsistencies, noise, missing data, outliers, high dimensionality, and imbalanced data. They noted that data preprocessing could be the best solution to address these issues and improve classifier performance. Following this, Alalawi and Alsuwat^33^ demonstrated that XGBoost outperformed Decision Tree (DT) after preprocessing raw data, detecting and removing outliers, and applying data balancing techniques. In another study, Subramani et al.^34^ utilized seven ML algorithms: Decision Tree (DT), K-Nearest Neighbors (KNN), Logistic Regression (LR), Naïve Bayes (NB), Random Forest (RF), Support Vector Machine (SVM), and XGB with GBDT feature selection to improve classifier performance. The proposed method achieved 96% accuracy, exceeding the performance of other existing models.

Furthermore, several studies have reported that high-dimensional datasets can be a major issue reducing the performance of machine learning (ML) techniques, as analyzing a large number of features requires significant memory and can lead to overfitting. Therefore, it is a wise decision to select features based on their importance, such as their weighting values, which can reduce redundant data, decrease processing time, and improve classifier performance. To address this, Garate-Escamila et al.^35^ proposed a dimensionality reduction method by using a feature selection technique to identify the most suitable and weighted features for classifying heart disease. They applied six ML classifiers: Decision Tree (DT), Gradient-boosted Tree (GBT), Logistic Regression (LR), Multilayer Perceptron (MLP), Naïve Bayes (NB), and Random Forests (RF) using two feature selection methods: Chi-square and principal component analysis (CHI-PCA). Among these, RF achieved 98.7% accuracy on the Cleveland dataset, 99.0% on the Hungarian dataset, and 99.4% on the Cleveland-Hungarian (CH) dataset.

In another study, Güllü et al.^36^ employed genetic and tabu search algorithms for feature selection, integrating Random Forest (RF), Adaboost, Bagging, Logitboost, and Support Vector Machine (SVM) models. This study showed a significant improvement in model performance (5.64%) considering combined datasets with optimization techniques. Another study conducted by Bhatt et al.^37^ employed k-model clustering with Random Forest (RF), Decision Tree (DT), MP, and XGBoost models, converting numerical data to categorical data and using GridSearchCV for parameter tuning. The study compared K-fold cross-validation, which demonstrated superior accuracy compared to models without cross-validation. In another study, Gupta and Seth^38^ presented a heart disease prediction model addressing the importance of feature selection process. They employed ML classifiers and a deep learning technique (Multi-Layer Perceptron (MLP)) for the classification and prediction of heart disease. The ML algorithms were optimized using hyperparameter tuning, and performance was evaluated based on prediction accuracy. Among the considered classification algorithms, the Random Forest (RF) achieved the best prediction accuracy (97.13%).

Following a similar objective, Dissanayake and Johar^39^ proposed a heart disease classification model that considers ten feature selection techniques (ANOVA, Chi-square, mutual information, ReliefF, forward feature selection, backward feature selection, exhaustive feature selection, recursive feature elimination, Lasso regression, and Ridge regression) and six ML classification methods (Decision Tree (DT), Random Forest (RF), Support Vector Machine (SVM), K-Nearest Neighbor (KNN), Logistic Regression (LR), and Gaussian naive Bayes). The classification results show that the backward feature selection technique with the DT classifier achieved the highest accuracy (88.52%), precision (91.30%), sensitivity (80.76%), and F-measure (85.71%). Additionally, Li et al.^40^ proposed an ML-based heart disease identification system developed with six classification algorithms, including Support Vector Machine (SVM), Logistic Regression (LR), Artificial Neural Network (ANN), K-Nearest Neighbor (KNN), Naïve Bayes (NB), and Decision Tree (DT). To enhance performance, they integrated several feature selection techniques to remove irrelevant and redundant features, such as Relief, Minimal Redundancy Maximal Relevance, Least Absolute Shrinkage and Selection Operator, and Local Learning. The experimental results indicated that using SVM with feature selection techniques significantly improves the system’s ability to identify heart disease.

Additionally, some researchers have addressed various data-related challenges in heart disease classification and prediction. For example, the research presented by^41^ demonstrates strategies for tackling limitations related to small data by utilizing surrogate data generated from original observations, implementing Neural Network models (Perceptron), and traditional machine learning models (Logistic Regression (LR), Decision Tree (DT), and Random Forest (RF)). This study showed that surrogate data improved accuracy by about 2% for traditional machine learning models, while evaluation results remained consistent with the original data. Furthermore, Louridi et al.^42^ focused on handling missing data using mean values, K-Nearest Neighbor (KNN), Multiple Imputations by Chained Equations (MICE), and Random Forest (RF) algorithms, and applied Nu SVM, Gradient Boosting regressor, Extreme Gradient Boosting, ADA Boost, ExtraTrees, LGBM, SGD, and stacking algorithms. The stacking algorithm achieved an accuracy of 93.85%, which offers potential benefits for physician diagnosis and data management.

In another study conducted by Abdellatif et al.^43^, the authors discussed the importance of optimization methods that significantly enhance the performance of ML models for cardiovascular disease (CVD) detection and severity level classification. They employed the Synthetic Minority Oversampling Technique (SMOTE) to address class imbalance issues and six ML classifiers (Support Vector Machine (SVM), K-Nearest Neighbor (KNN), Logistic Regression (LR), SGD, ET, XGBoost) to determine the patient’s status. They also utilized Hyperparameter Optimization (HPO) to identify the best hyperparameters for the chosen classifiers. The results indicated that SMOTE and the Extra Trees (ET) optimization technique achieved superior outcomes.

Additionally, several studies experimented with neural networks and deep learning methods for heart disease classification and prediction. One study conducted by Hossam Meshref^44^ reported that ANN achieved the highest accuracy of 84.25%, although both ANN and SVM showed low learning speeds. Furthermore, ANN tends to perform better on linearly separable data, while SVM excels at maximizing hyperplane margins, which improves both accuracy and transparency. In another study, Abdulwahab Ali Almazroi^45^ compared four machine learning techniques: Decision Tree (DT), Random Forest (RF), Artificial Neural Network (ANN), and Support Vector Machine (SVM). Among these, ANN showed comparatively lower performance, while DT achieved good accuracy (80%). Additionally, Kim et al.^46^ conducted a meta-analysis of machine learning approaches and suggested that deep learning models may not perform optimally with limited data due to overfitting issues. This study also demonstrated that support vector machine and boosting models showed better results for cardiovascular disease and stroke risk classification; however, there were limitations related to the correlation of clinical data. Moreover, some research has focused on using deep learning methods to analyze ECG signals for the automatic detection and classification of cardiovascular diseases. For instance, Zhang et al.^47^ proposed a model incorporating Convolutional Neural Networks (CNN), trained on 259,789 ECG signals from a tertiary care hospital and tested on 18,018 ECG signals. They classified the ECG signals into normal and abnormal categories. Their model achieved 95% overall accuracy.

With a similar goal, another study^48^ addressed the key challenge in ECG signal analysis by handling irregularities that can affect accurate detection of heart disease. Therefore, they proposed an efficient approach to classify ECG signals with high accuracy, incorporating several ML classifiers (Decision Tree (DT), Random Forests (RF), and Gradient-Boosted Trees (GDB)). Their proposed method was tested on the baseline MIT-BIH Arrhythmia and MIT-BIH Supraventricular Arrhythmia databases. The results show that, for binary classification, the proposed model achieved an overall accuracy of 96.75% using the GDB tree algorithm and 97.98% with the RF classifier. Additionally, for multi-class classification, the proposed model achieved an accuracy of 98.03%, using RF and Gradient Boosting Tree algorithms.

Recently, various data mining tools have been used with machine learning classifiers to analyze healthcare data and uncover useful patterns. As data mining tools facilitate the task of classifying heart disease, Tougui et al.^49^ applied six machine learning techniques (Logistic Regression (LR), Support Vector Machine (SVM), K-Nearest Neighbor (KNN), Artificial Neural Network (ANN), Naïve Bayes (NB), and Random Forest (RF)) using six data mining tools (Orange, Weka, RapidMiner, Knime, Matlab, and Scikit-Learn). The performance of each tool and ML technique was measured through accuracy, sensitivity, and specificity scores. Among the selected tools, Matlab was the best, with Matlab’s Artificial Neural Network model being the top ML technique.

Moreover, the comprehensive review of several existing literature discussed in this section highlights several limitations in heart disease classification research. Among several limitations, the most frequent issues are:

- Studies such as^41,42^ address challenges like missing data and small datasets; however, they do not focus on standardized data preprocessing or different feature selection combinations.
- While ensemble techniques^28,29^ have been applied to heart disease classification, relying exclusively on these methods may fail to uncover deeper insights or patterns in the classified data.

To address these limitations, this study analyzes a balanced dataset using different combinations of advanced feature selection methods and ensemble techniques to compare the performance of various machine learning models. The ultimate goal is to identify the model that performs better under specific preprocessing strategies and has strong potential for improving predictive performance.

## Methodology

**Fig. 1:**
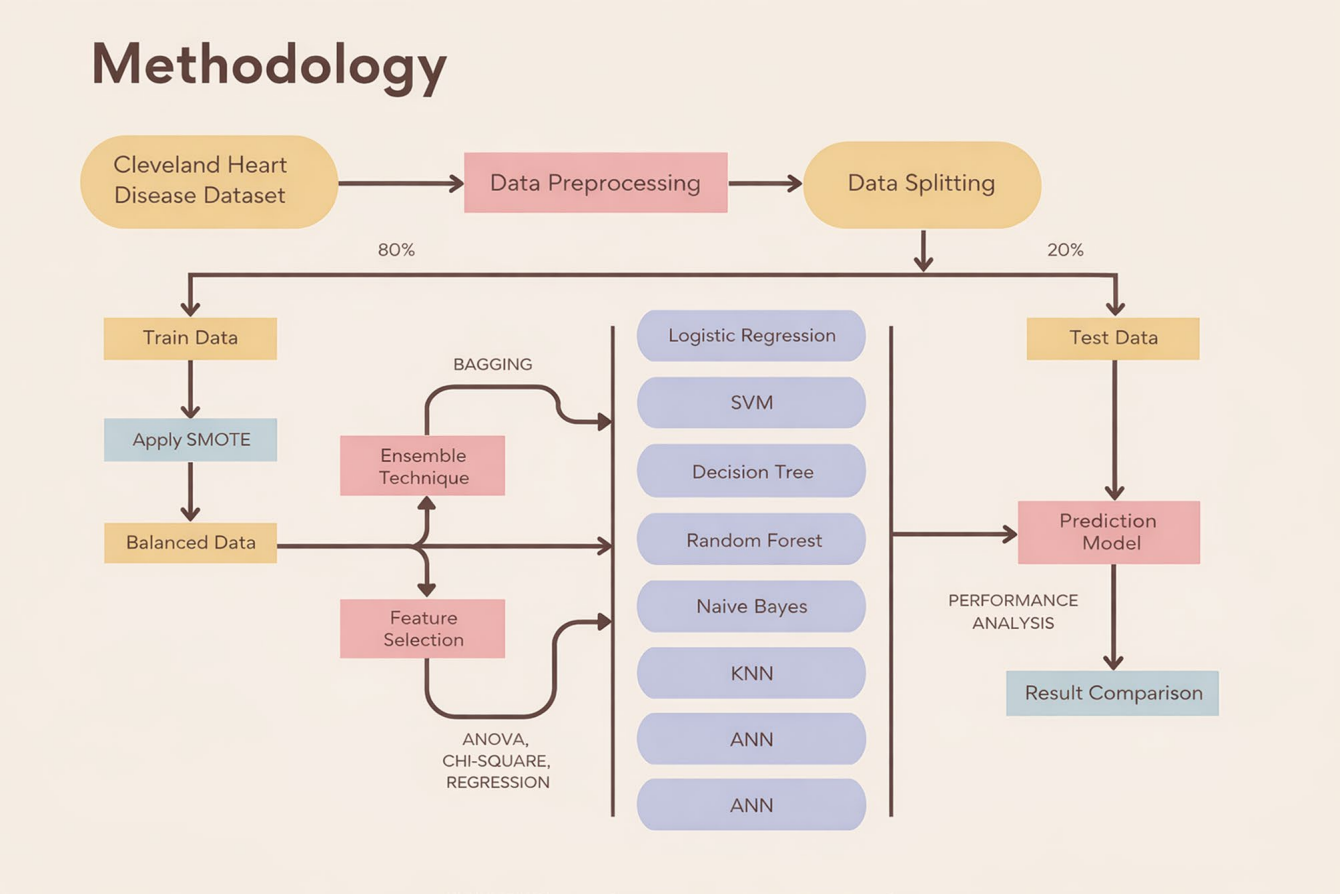
Heart disease classification methodology.

This research aims to evaluate machine learning models for heart disease classification using feature selection and ensemble-based preprocessing techniques. This section discusses data collection, dataset description, pre-processing, feature engineering, and relevant machine learning methods. It includes a block diagram, evaluation metrics, and the study’s techniques and methodology. The proposed framework is illustrated in Fig. 1.

Figure 1 illustrates the proposed methodology, detailing the workflow from dataset preparation to result analysis. The process comprises six main steps:

- Dataset Collection and Preprocessing (Sect.  3.1): In this step, the dataset is collected, pre-processed to handle missing or inconsistent data, and subsequently split into training (80%) and testing (20%) sets. To address class imbalance in the training data, the Synthetic Minority Oversampling Technique (SMOTE) is applied.
- Feature Selection (Sect.  3.2): Feature selection techniques are employed to identify the most relevant features, reducing dimensionality and improving model efficiency. The resulting dataset is refined for the subsequent steps.
- Ensemble Techniques (Sect.  3.3): Bagging-based ensemble techniques are implemented to generate random subsets of the dataset. This process creates multiple training datasets that enhance model diversity and reduce variance.
- Model Training (Sect.  3.4): Seven machine learning models, Logistic Regression (LR), Decision Tree (DT), Random Forest (RF), Naive Bayes (NB), Support Vector Machine (SVM), Artificial Neural Networks (ANN), and K-Nearest Neighbors (KNN), are trained in three stages:
  - First, models are trained on the balanced training dataset and evaluated on the testing dataset.
  - Second, models are trained using the feature-selected training dataset and evaluated on the testing dataset.
  - Third, bagging-based ensemble models are trained on bootstrapped samples of the training data, and performance is evaluated on the unchanged testing dataset.
- Model Evaluation (Sect.  3.5): Each model is evaluated using the test dataset, applying appropriate performance metrics to assess predictive accuracy, precision, recall, and F1-score.
- Result Analysis (Sect.  3.6): The results from all experiments are analyzed to compare the performance of the models. Insights are drawn to achieve the best performance metrics and dataset preparation strategies.

### Dataset collection and preprocessing

This study utilizes the BRFSS 2020 Heart Disease Dataset (BRFSS 2020 Heart Disease Dataset: https://zenodo.org/records/15364962.), which is a publicly available dataset obtained from the Zenodo data repository for model development and analysis. We used this dataset for its curated, pre-processed structure, which supports reproducibility and consistency with prior studies. Although newer BRFSS datasets are available from the CDC, our study focuses on the 2020 BRFSS Heart Disease dataset, as it has been widely used in existing studies, while the 2024 dataset is relatively recent and less established in the literature. This dataset is derived from the 2020 Behavioral Risk Factor Surveillance System (BRFSS) and contains self-reported clinical, demographic, and lifestyle information related to cardiovascular health. It includes 17 attributes such as smoking status, alcohol consumption, history of stroke, asthma, skin cancer, and other cardiovascular risk indicators. The dataset was developed for research purposes and is suitable for supervised learning and cardiovascular risk prediction tasks. All experiments in this study were conducted exclusively using this dataset. The attributes of the selected dataset are summarized in Table 1.

**Table 1:** Attributes of the dataset.

Input Features	Type	Category	Description	Input Features	Type	Category	Description
Heart Disease	Nominal	2	Presence of heart disease (Yes, No)	Age Category	Nominal	13	Age category (e.g., 55–59, 80 of older)
BMI	Numeric	–	Body Mass Index	Race	Nominal	–	Race of the individual
Smoking	Nominal	2	Smoking status (Yes, No)	Diabetic	Nominal	2	Diabetic status (Yes, No)
Alcohol Drinking	Nominal	2	Alcohol Drinking status (Yes, No)	Physical Activity	Nominal	2	Participation in physical activities of exercises (Yes, No)
Stroke	Nominal	2	History of stroke (Yes, No)	Gen Health	Nominal	5	General health condition (Very good, Fair, Good, Poor, Excellent)
Physical Health	Numeric	–	Number of days of poor physical health in the past 30 days	Sleep time	Numeric	–	Average hours of sleep per day
Mental Health	Numeric	–	Number of days of poor mental health in the past 30 days	Asthma	Nominal	2	Asthma status (Yes, No)
Diff Walking	Nominal	2	Difficulty walking of climbing stairs (Yes, No)	Kidney Disease	Nominal	2	Presence of kidney disease (Yes, No)
Sex	Nominal	2	Gender (Female, Male)	Skin Cancer	Nominal	2	History of skin cancer (Yes, No)

#### Data pre-processing

Using real-world data to validate machine learning models is relatively challenging due to data ambiguity. These issues often hinder optimal machine learning (ML) results, for example, outliers, missing values, and inherent inconsistencies. However, these problems can be addressed by applying data preprocessing techniques according to data type. This process carefully prepares the data for analysis, unlocking its full potential for accurate predictions and building confidence in the research process. Therefore, we implemented several data pre-processing techniques to reduce ambiguities and enhance the quality of the selected dataset as follows:

1. Missing Value Intervention: We performed data pre-processing by addressing missing values in the dataset, which is common in real-world data. Depending on the data type, we used mean/median imputation strategies for numerical data and mode imputation for categorical data. This careful approach ensured data reliability.
2. Feature Transformation: The raw data included various feature types, such as strings and numbers. The transformation was performed using feature engineering to ensure all features were converted to numeric values when necessary.
3. Outlier Eviction: Outliers, which are known as different data points that deviate significantly from the norm, can introduce noise and hinder model performance. These outliers were identified and eliminated from the dataset to improve data quality.

#### Data splitting and balancing

Data splitting: Data splitting allows dividing a dataset into distinct groups to facilitate model training and evaluation. The data of the selected dataset is randomly partitioned into two subsets:

- *Training Data*: This subset comprises the majority of the data, which is used to train the selected seven machine learning models. It serves as the basis for the models to learn patterns and relationships within the data.
- *Testing Data*: A smaller subset used to evaluate the performance of trained models. Testing data is unseen during training, ensuring an unbiased assessment of the model’s predictive ability.

In this study, the dataset was split into an 80:20 ratio, where 80% for training and 20% for testing. This common splitting ratio ensures sufficient training data while maintaining adequate data for reliable evaluation.

Balancing the Dataset: The dataset showed class imbalance, as a few classes were underrepresented relative to others. Class imbalance can negatively impact model performance by biasing predictions toward the majority class. To address this issue, the Synthetic Minority Oversampling Technique (SMOTE) was applied. SMOTE is an oversampling method that creates synthetic samples for the minority class. It does this by interpolating new samples between existing ones based on their closest neighbors in the feature space. By balancing the dataset, SMOTE improves the model’s ability to predict true outcomes for all classes, reduces bias, and boosts overall performance. This balanced training dataset was then used to train machine learning models.

### Feature selection

Feature selection is crucial in machine learning for improving model performance and reducing feature dimensionality by identifying the features with higher statistical relevance in a dataset. It enhances accuracy, minimizes overfitting, and simplifies the model for better understanding. In this study, we implemented three feature selection techniques: ANOVA, Chi-Square Test, and Regression Analysis to determine key features. For each method, features were ranked by importance. Selecting the appropriate features reduces dimensionality, improves interpretability, and increases data quality^50–52^. Feature normalization was then applied to these selected features for robust analysis.

#### ANOVA (Analysis of Variance)

ANOVA is a statistical method used to identify significant features by comparing differences between different groups. This process involves:

- Hypothesis Testing: Testing the null hypothesis (no difference in means) against the alternative hypothesis (significant difference in means).
- F-Statistic Calculation: Determining the ratio of between-group variance to within-group variance for each feature.
- P-Value Evaluation: Features with p-values less than 0.05 are considered significant.

**Fig. 2:**
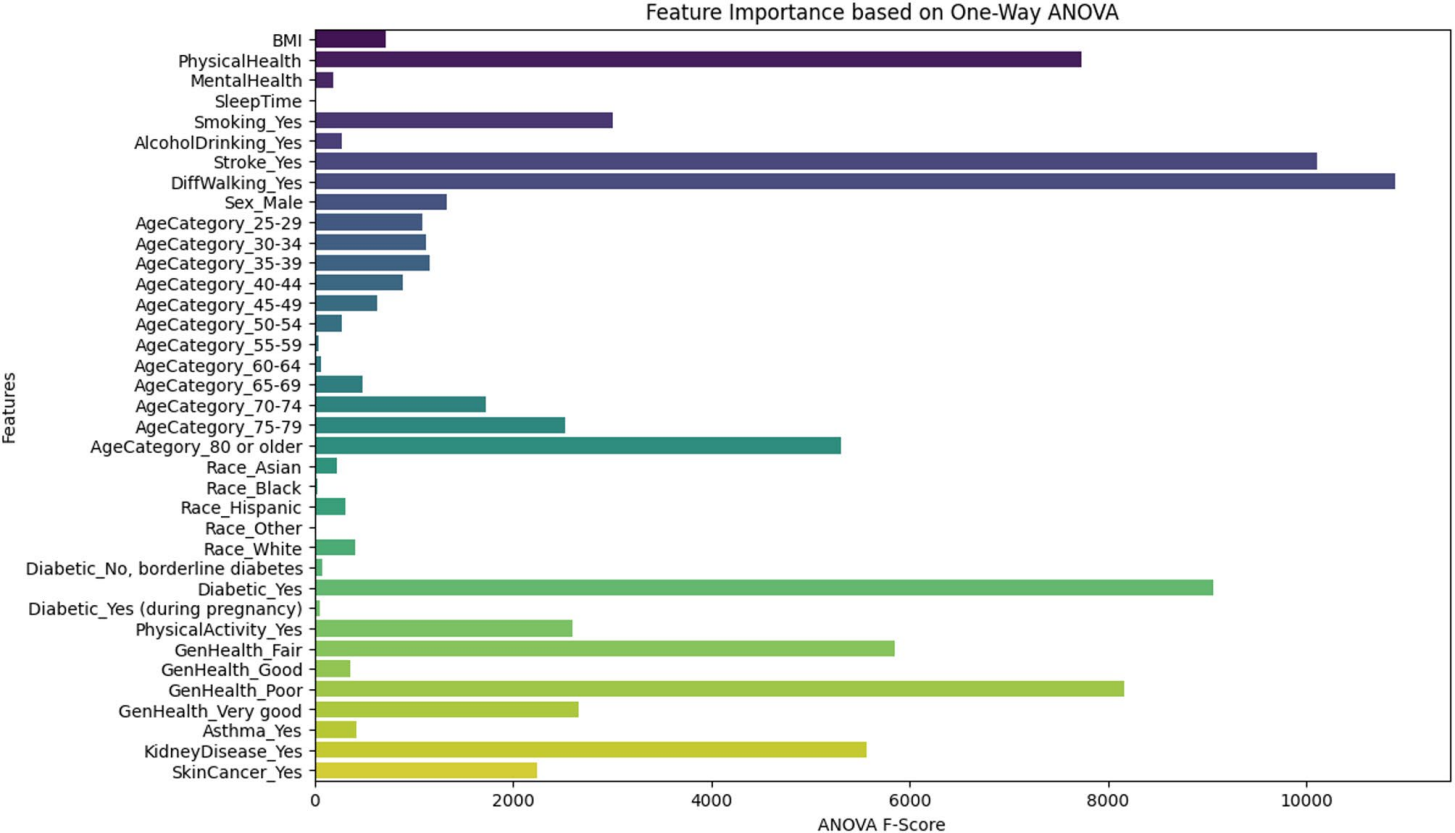
Feature importance using the ANOVA test.

Figure 2 presents the ANOVA test results for all features, including their F-scores and feature importance. This figure indicates that [PhysicalHealth, Stroke_Yes, DiffWalking_Yes, AgeCategory_80 or older, Diabetic_Yes, GenHealth_Fair, GenHealth_Poor, KidneyDisease_Yes] are the higher relevance features according to the ANOVA test.

#### Chi-Square Test

The Chi-Square test evaluates the relationship between categorical features and the target variable by measuring the deviation of observed values from expected values under the null hypothesis. This process involves:

- Contingency Table Creation: Frequency distribution of feature values across target classes.
- Chi-Square Statistic Calculation: Quantifying the association strength.
- P-Value Evaluation: The p-value determines the statistical significance of the association. For this study, we considered features with p-values < 0.05 to ensure that only features meeting the chosen significance threshold were retained for further analysis. All experiments and reported results were recomputed using this corrected criterion.

**Fig. 3:**
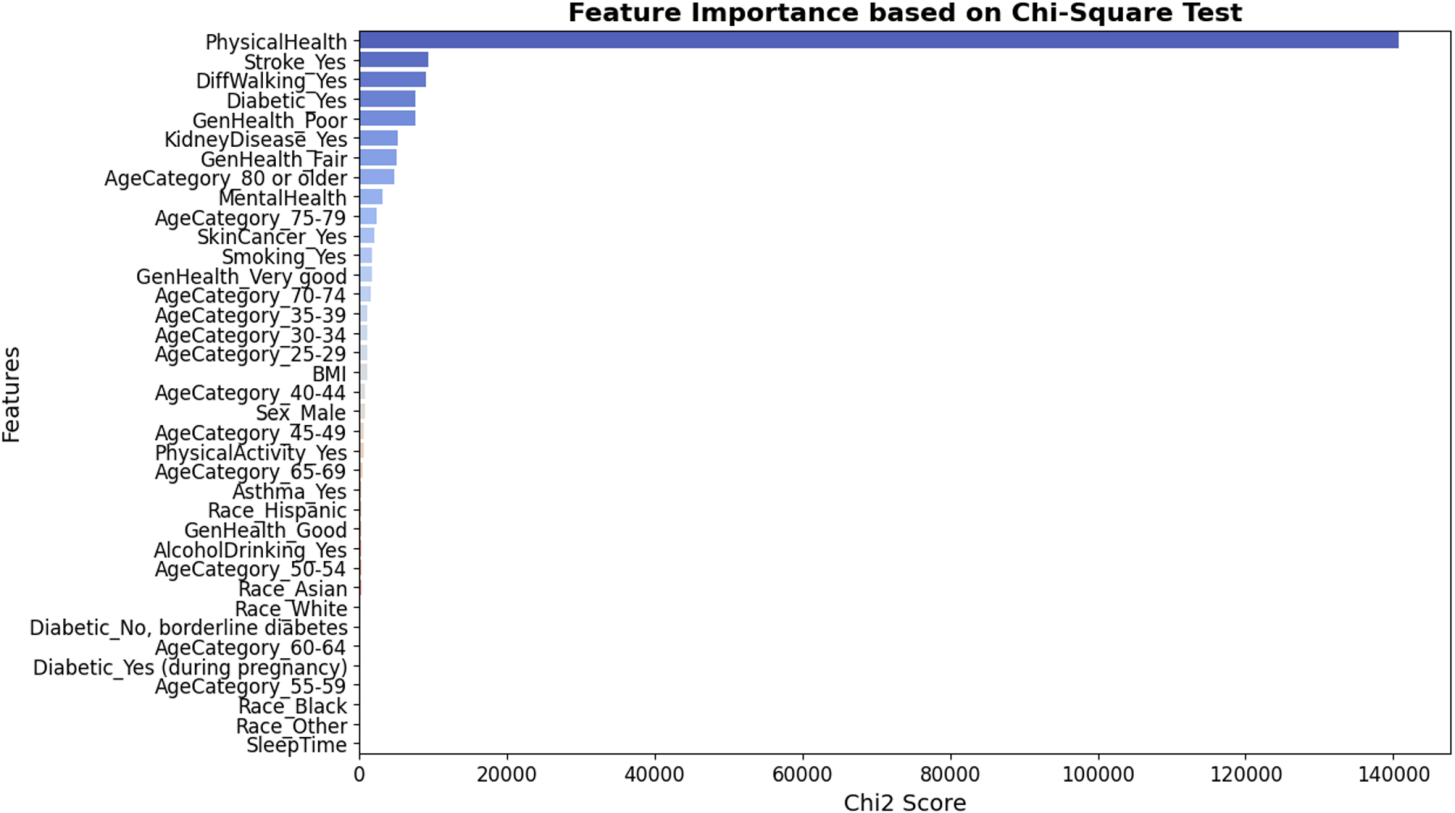
Feature Importance using Chi-Square test.

Figure 3 displays the features ranked by their importance using the Chi-Square test. This figure indicate that [AgeCategory_35–39, AgeCategory_30–34, AgeCategory_25–29, BMI, AgeCategory_40–44, Sex_Male, AgeCategory_45–49, PhysicalActivity_Yes, AgeCategory_65–69, Asthma_Yes, Race_Hispanic, GenHealth_Good, AlcoholDrinking_Yes, AgeCategory_50–54, Race_Asian, Race_White, Diabetic_No, borderline diabetes, AgeCategory_60–64, Diabetic_Yes (during pregnancy), AgeCategory_55–59, Race_Black, Race_Other, and SleepTime] are identified as the most significant features based on their Chi-Square values.

#### Regression analysis

Regression analysis is a predictive modeling technique that estimates the relationship between features and the target variable. In the context of feature selection, regression analysis identifies relevant features that contribute to the model’s predictive performance. The process involves:

- Coefficient Analysis: Examining feature coefficients to assess their impact on the target variable.
- Significance Testing: Features with statistically significant coefficients are prioritized for selection.
- Performance Validation: Verifying the effectiveness of selected features through model evaluation metrics.

**Fig. 4:**
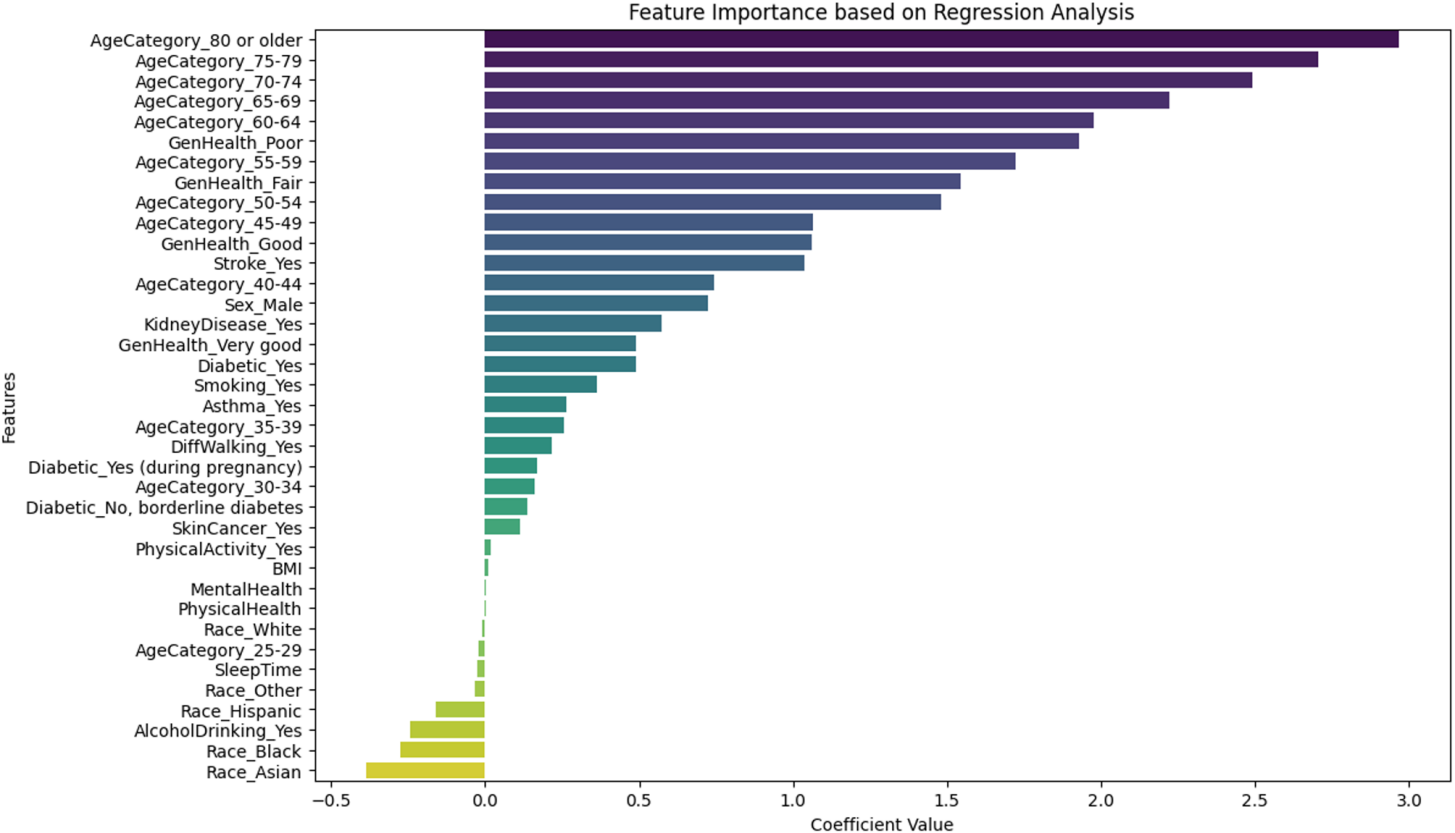
Feature Importance using Regression analysis.

Figure 4 shows the feature analysis result using regression analysis. It indicates that features with higher statistical relevance are included [AgeCategory_80 or older, AgeCategory_75–79, AgeCategory_70–74, AgeCategory_65–69, AgeCategory_60–64, GenHealth_Poor, AgeCategory_55–59, GenHealth_Fair, AgeCategory_50–54, AgeCategory_45–49, GenHealth_Good, Stroke_Yes, AgeCategory_40–44, Sex_Male, KidneyDisease_Yes, GenHealth_Very good, Diabetic_Yes, Smoking_Yes, Asthma_Yes, AgeCategory_35–39, DiffWalking_Yes, Diabetic_Yes (during pregnancy), AgeCategory_30–34, Diabetic_No, borderline diabetes, SkinCancer_Yes, PhysicalActivity_Yes, BMI].

#### Feature normalization

To enhance model performance and interpretability, features were normalized and refined using eight (8) distinct combinations of the selected three feature analysis techniques (as described in Sect. 3.2.1, 3.2.2, and 3.2.3). These combinations were derived from the authors’ empirical observations and supported by insights from the literature on feature normalization^51,52^. The eight distinct combinations are: {ANOVA ∪ Chi-Square}, {ANOVA ∪ Regression Analysis}, {Chi-Square ∪ Regression Analysis}, {ANOVA ∪ Chi-Square ∪ Regression Analysis}, {ANOVA ∩ Chi-Square}, {ANOVA ∩ Regression Analysis}, {Chi-Square ∩ Regression Analysis}, and {ANOVA ∩ Chi-Square ∩ Regression Analysis}.

**Fig. 5:**
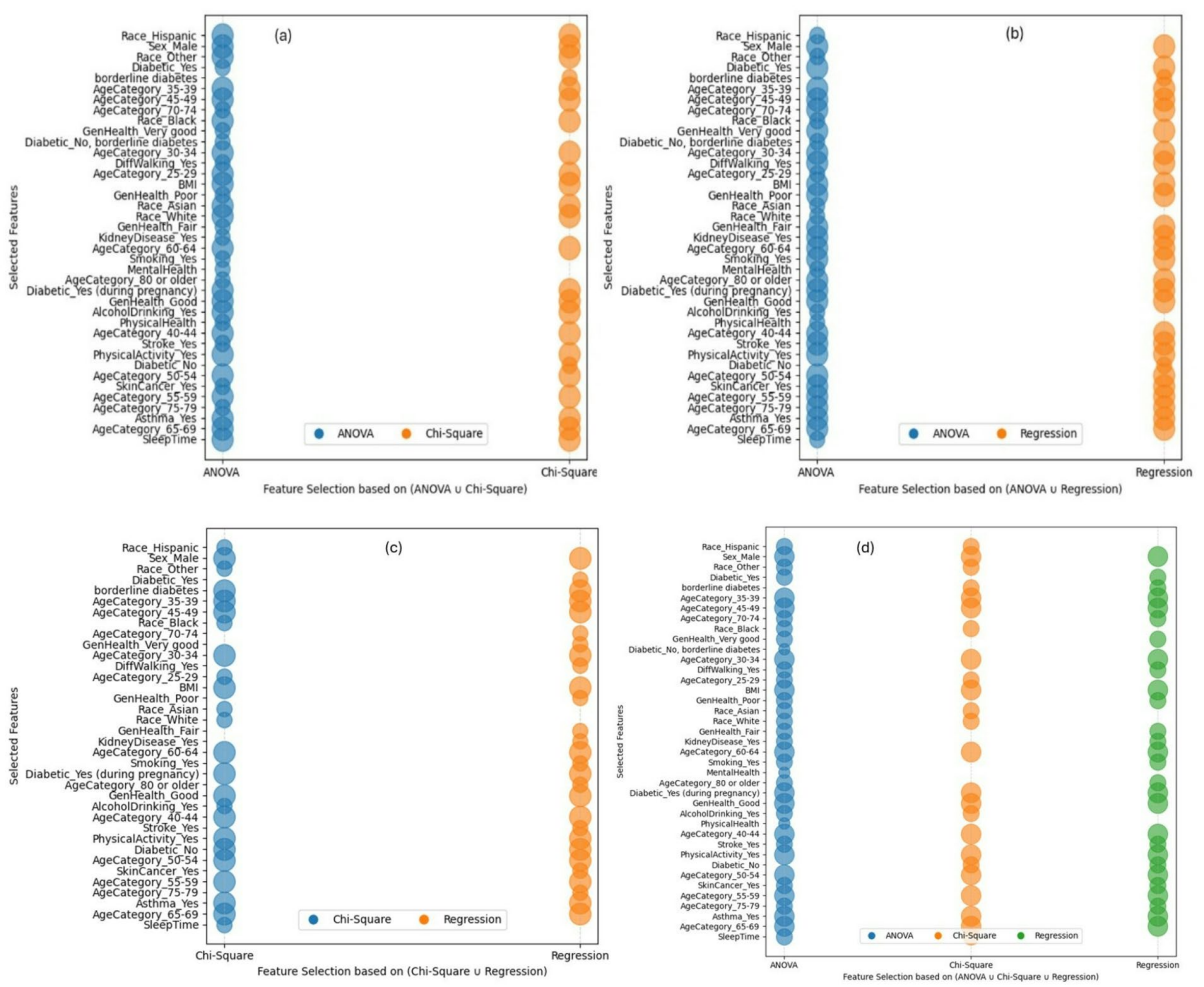
Classified features in the combination of (**a**) {ANOVA ∪ Chi-Square}, (**b**) {ANOVA ∪ Regression Analysis}, (**c**) {Chi-Square ∪ Regression Analysis}, and (**d**) {ANOVA ∪ Chi-Square ∪ Regression Analysis}.

**Fig. 6:**
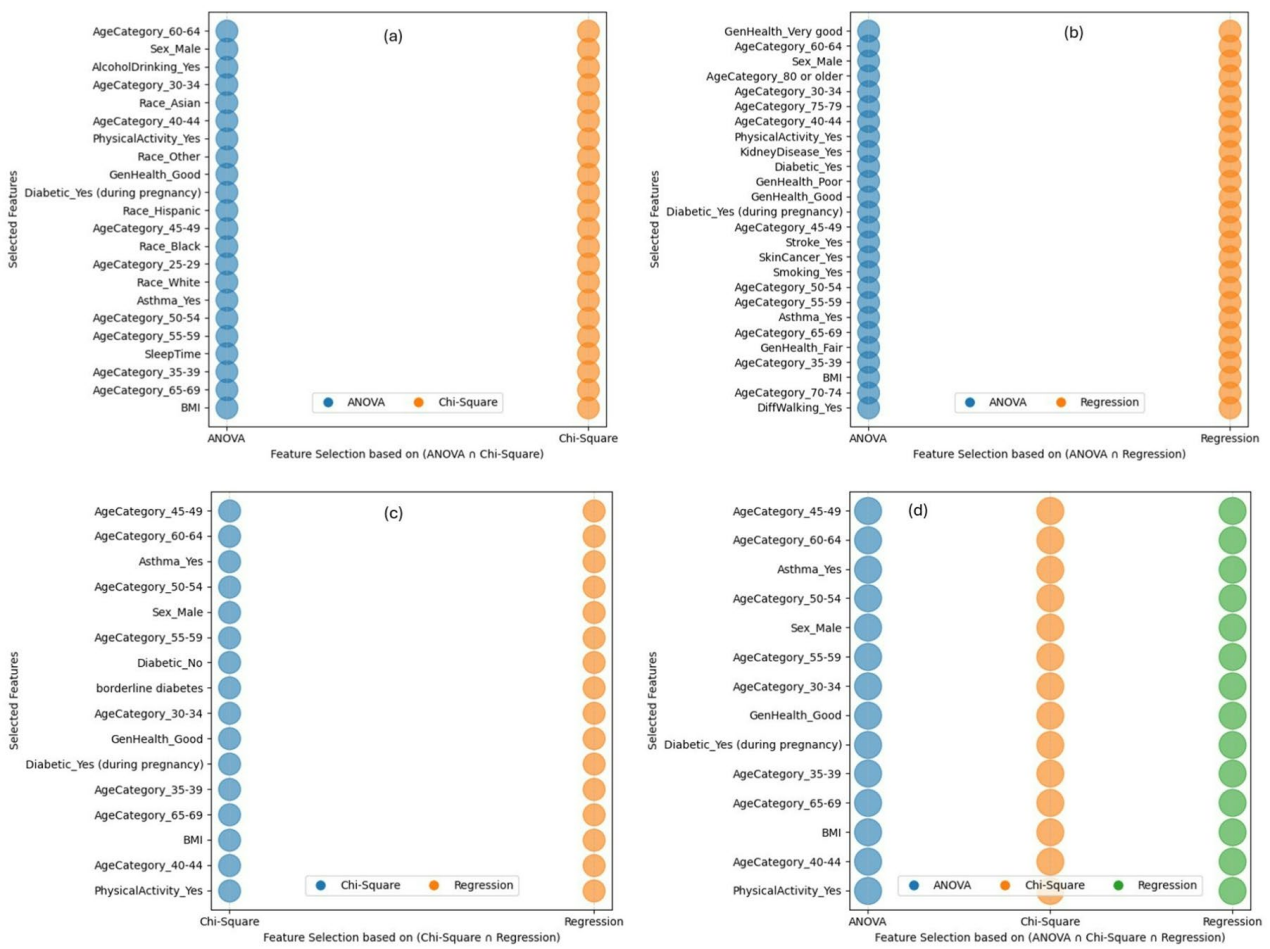
Classified features in the combination of (**a**) {ANOVA ∩ Chi-Square}, (**b**) {ANOVA ∩ Regression Analysis}, (**c**) {Chi-Square ∩ Regression Analysis}, and (**d**) {ANOVA ∩ Chi-Square ∩ Regression Analysis}.

- Union of (ANOVA and Chi-Square): By focusing on features identified as significant by both ANOVA and Chi-Square tests, this combination emphasizes both numerical and categorical variables with high relevance to the target variable. Figure 5 (a) shows the most important classified features obtained from this feature normalization or combination including [‘Race_Hispanic’, ‘Sex_Male’, ‘Race_Other’, ‘Diabetic_Yes’, ‘borderline diabetes’, ‘AgeCategory_35–39’, ‘AgeCategory_45–49’, ‘AgeCategory_70–74’, ‘Race_Black’, ‘GenHealth_Very good’, ‘Diabetic_No, borderline diabetes’, ‘AgeCategory_30–34’, ‘DiffWalking_Yes’, ‘AgeCategory_25–29’, ‘BMI’, ‘GenHealth_Poor’, ‘Race_Asian’, ‘Race_White’, ‘GenHealth_Fair’, ‘KidneyDisease_Yes’, ‘AgeCategory_60–64’, ‘Smoking_Yes’, ‘MentalHealth’, ‘AgeCategory_80 or older’, ‘Diabetic_Yes (during pregnancy)’, ‘GenHealth_Good’, ‘AlcoholDrinking_Yes’, ‘PhysicalHealth’, ‘AgeCategory_40–44’, ‘Stroke_Yes’, ‘PhysicalActivity_Yes’, ‘Diabetic_No’, ‘AgeCategory_50–54’, ‘SkinCancer_Yes’, ‘AgeCategory_55–59’, ‘AgeCategory_75–79’, ‘Asthma_Yes’, ‘AgeCategory_65–69’, ‘SleepTime’].
- Union of (ANOVA and Regression Analysis): This approach combines the strengths of ANOVA and Regression analysis, ensuring the inclusion of features with both statistical significance and predictive power. Figure 5 (b) shows the classified features determined from this feature normalization or combination include [‘Race_Hispanic’, ‘Sex_Male’, ‘Race_Other’, ‘Diabetic_Yes’, ‘borderline diabetes’, ‘AgeCategory_35–39’, ‘AgeCategory_45–49’, ‘AgeCategory_70–74’, ‘Race_Black’, ‘GenHealth_Very good’, ‘Diabetic_No, borderline diabetes’, ‘AgeCategory_30–34’, ‘DiffWalking_Yes’, ‘AgeCategory_25–29’, ‘BMI’, ‘GenHealth_Poor’, ‘Race_Asian’, ‘Race_White’, ‘GenHealth_Fair’, ‘KidneyDisease_Yes’, ‘AgeCategory_60–64’, ‘Smoking_Yes’, ‘MentalHealth’, ‘AgeCategory_80 or older’, ‘Diabetic_Yes (during pregnancy)’, ‘GenHealth_Good’, ‘AlcoholDrinking_Yes’, ‘PhysicalHealth’, ‘AgeCategory_40–44’, ‘Stroke_Yes’, ‘PhysicalActivity_Yes’, ‘Diabetic_No’, ‘AgeCategory_50–54’, ‘SkinCancer_Yes’, ‘AgeCategory_55–59’, ‘AgeCategory_75–79’, ‘Asthma_Yes’, ‘AgeCategory_65–69’, ‘SleepTime’].
- Union of (Chi-Square and Regression Analysis): This process combines the features obtained from ANOVA and Regression Analysis to ensure the inclusion of the features with higher statistical relevance. Figure 5 (c) shows the classified features determined from this feature normalization or combination including [‘Race_Hispanic’, ‘Sex_Male’, ‘Race_Other’, ‘Diabetic_Yes’, ‘borderline diabetes’, ‘AgeCategory_35–39’, ‘AgeCategory_45–49’, ‘Race_Black’, ‘AgeCategory_70–74’, ‘GenHealth_Very good’, ‘AgeCategory_30–34’, ‘DiffWalking_Yes’, ‘AgeCategory_25–29’, ‘BMI’, ‘GenHealth_Poor’, ‘Race_Asian’, ‘Race_White’, ‘GenHealth_Fair’, ‘KidneyDisease_Yes’, ‘AgeCategory_60–64’, ‘Smoking_Yes’, ‘Diabetic_Yes (during pregnancy)’, ‘AgeCategory_80 or older’, ‘GenHealth_Good’, ‘AlcoholDrinking_Yes’, ‘AgeCategory_40–44’, ‘Stroke_Yes’, ‘PhysicalActivity_Yes’, ‘Diabetic_No’, ‘AgeCategory_50–54’, ‘SkinCancer_Yes’, ‘AgeCategory_55–59’, ‘AgeCategory_75–79’, ‘Asthma_Yes’, ‘AgeCategory_65–69’, ‘SleepTime’].
- Union of (ANOVA, Chi-Square, and Regression Analysis): This combination aims to capture a comprehensive set of features obtained from the selected three feature selection methods to identify feature subsets suitable for classification. The classified features (as shown in Fig. 5 (d)) obtained from this feature normalization or combination are: [‘Race_Hispanic’, ‘Sex_Male’, ‘Race_Other’, ‘Diabetic_Yes’, ‘borderline diabetes’, ‘AgeCategory_35–39’, ‘AgeCategory_45–49’, ‘AgeCategory_70–74’, ‘Race_Black’, ‘GenHealth_Very good’, ‘Diabetic_No, borderline diabetes’, ‘AgeCategory_30–34’, ‘DiffWalking_Yes’, ‘AgeCategory_25–29’, ‘BMI’, ‘GenHealth_Poor’, ‘Race_Asian’, ‘Race_White’, ‘GenHealth_Fair’, ‘KidneyDisease_Yes’, ‘AgeCategory_60–64’, ‘Smoking_Yes’, ‘MentalHealth’, ‘AgeCategory_80 or older’, ‘Diabetic_Yes (during pregnancy)’, ‘GenHealth_Good’, ‘AlcoholDrinking_Yes’, ‘PhysicalHealth’, ‘AgeCategory_40–44’, ‘Stroke_Yes’, ‘PhysicalActivity_Yes’, ‘Diabetic_No’, ‘AgeCategory_50–54’, ‘SkinCancer_Yes’, ‘AgeCategory_55–59’, ‘AgeCategory_75–79’, ‘Asthma_Yes’, ‘AgeCategory_65–69’, ‘SleepTime’].

- Intersection of (ANOVA and Chi-Square): This combination of features allows us to determine the most common features that appear on both ANOVA and Chi-Square analysis to support improved classification and prediction performance. Figure 6 (a) shows the classified common features from both ANOVA and Chi-Square analysis including [‘AgeCategory_60–64’, ‘Sex_Male’, ‘AlcoholDrinking_Yes’, ‘AgeCategory_30–34’, ‘Race_Asian’, ‘AgeCategory_40–44’, ‘PhysicalActivity_Yes’, ‘Race_Other’, ‘GenHealth_Good’, ‘Diabetic_Yes (during pregnancy)’, ‘Race_Hispanic’, ‘AgeCategory_45–49’, ‘Race_Black’, ‘AgeCategory_25–29’, ‘Race_White’, ‘Asthma_Yes’, ‘AgeCategory_50–54’, ‘AgeCategory_55–59’, ‘SleepTime’, ‘AgeCategory_35–39’, ‘AgeCategory_65–69’, ‘BMI’].
- Intersection of (ANOVA and Regression Analysis): This combination determines the most influential features by selecting the common features from both ANOVA and Regression Analysis. Figure 6 (b) shows the classified features from ANOVA and Regression Analysis that obtained twenty five features including [‘GenHealth_Very good’, ‘AgeCategory_60–64’, ‘Sex_Male’, ‘AgeCategory_80 or older’, ‘AgeCategory_30–34’, ‘AgeCategory_75–79’, ‘AgeCategory_40–44’, ‘PhysicalActivity_Yes’, ‘KidneyDisease_Yes’, ‘Diabetic_Yes’, ‘GenHealth_Poor’, ‘GenHealth_Good’, ‘Diabetic_Yes (during pregnancy)’, ‘AgeCategory_45–49’, ‘Stroke_Yes’, ‘SkinCancer_Yes’, ‘Smoking_Yes’, ‘AgeCategory_50–54’, ‘AgeCategory_55–59’, ‘Asthma_Yes’, ‘AgeCategory_65–69’, ‘GenHealth_Fair’, ‘AgeCategory_35–39’, ‘BMI’, ‘AgeCategory_70–74’, ‘DiffWalking_Yes’].
- Intersection of (Chi-Square and Regression Analysis): This combination finds the most significant features by choosing the common features from both Chi-Square and Regression Analysis results. Figure 6 (c) shows the classified features including [‘AgeCategory_45–49’, ‘AgeCategory_60–64’, ‘Asthma_Yes’, ‘AgeCategory_50–54’, ‘Sex_Male’, ‘AgeCategory_55–59’, ‘Diabetic_No’, ‘borderline diabetes’, ‘AgeCategory_30–34’, ‘GenHealth_Good’, ‘Diabetic_Yes (during pregnancy)’, ‘AgeCategory_35–39’, ‘AgeCategory_65–69’, ‘BMI’, ‘AgeCategory_40–44’, ‘PhysicalActivity_Yes’].
- Intersection of (ANOVA, Chi-Square, and Regression Analysis): To choose the appropriate feature subset for the classification, this combination aims to represent a complete set of common features derived from the three feature selection techniques: ANOVA, Chi-Square, and Regression Analysis. Figure 6 (d) shows the classified features commonly appeared in three selected feature selection methods including [‘AgeCategory_45–49’, ‘AgeCategory_60–64’, ‘Asthma_Yes’, ‘AgeCategory_50–54’, ‘Sex_Male’, ‘AgeCategory_55–59’, ‘AgeCategory_30–34’, ‘GenHealth_Good’, ‘Diabetic_Yes (during pregnancy)’, ‘AgeCategory_35–39’, ‘AgeCategory_65–69’, ‘BMI’, ‘AgeCategory_40–44’, ‘PhysicalActivity_Yes’].

### Ensemble technique

**Fig. 7:**
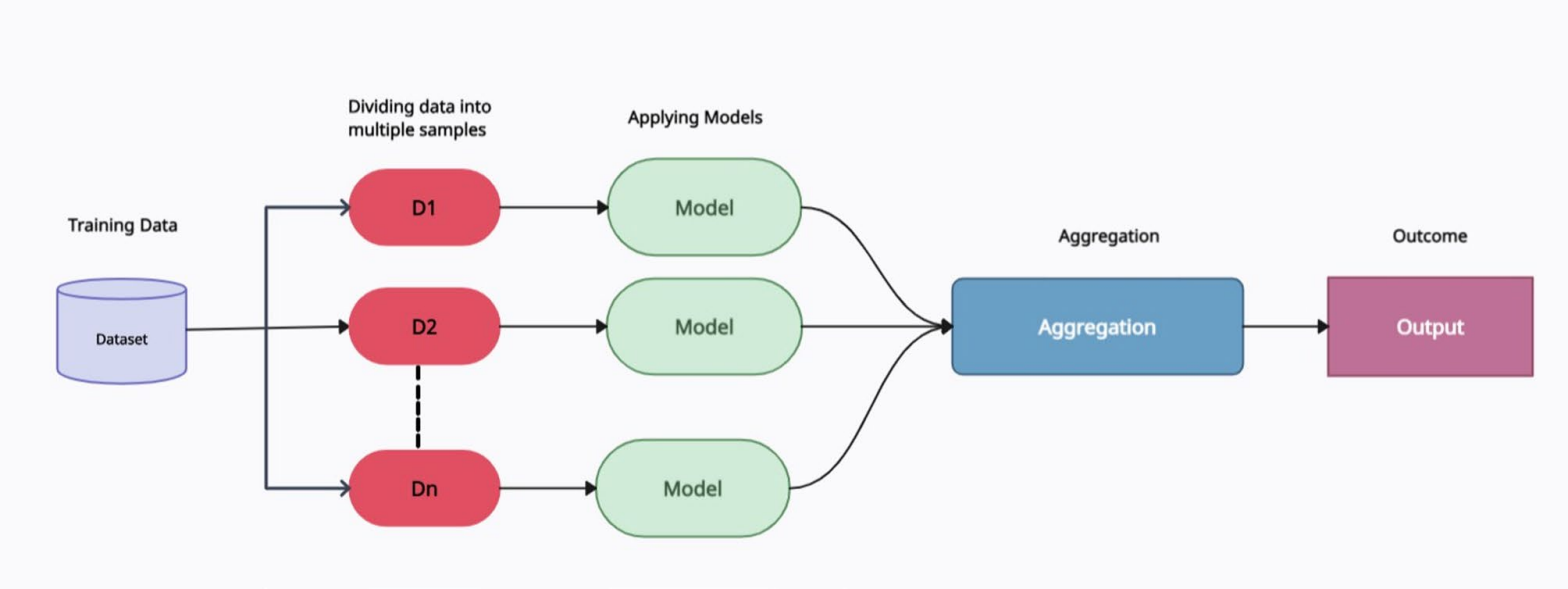
Ensemble learning (bagging technique).

Ensemble techniques involve combining the predictions of multiple models to achieve a more accurate and reliable overall model. Specifically, the bagging technique is used in this study. The bagging process is shown in Fig. 7, indicating how multiple models are aggregated to improve prediction accuracy. This illustration emphasizes the formulation of diverse subsets from the original dataset and their subsequent combination.

Generally, bagging is an ensemble learning technique that aims to reduce variance and prevent overfitting by generating multiple versions of a training dataset and using them to train several models. The steps involved in bagging include:

- Bootstrap Sampling: Multiple subsets of the training dataset are created using bootstrap sampling. This involves randomly selecting samples from the original dataset with replacement, resulting in different subsets that may contain duplicate samples.
- Model Training: Each bootstrap sample is used to train a separate model of the same type (e.g., decision trees). Together, these models form an ensemble, with each model trained on a different bootstrap sample.
- Aggregation: The predictions from all models in the ensemble are combined to produce a final prediction. For classification tasks, this is performed through majority voting to compute the overall outcome. For regression tasks, the final prediction is generally the average of all individual model predictions.

### Machine learning models training

After data preprocessing, seven different machine learning (ML) algorithms: Logistic Regression (LR), Decision Tree (DT), Random Forest (RF), Naive Bayes (NB), Support Vector Machine (SVM), Artificial Neural Networks (ANN), and K-Nearest Neighbors (KNN) are applied for further analysis. The details of these algorithms are as follows:

#### Logistic Regression (LR)

Logistic Regression (LR) is a statistical classification method that estimates the probability of a binary outcome using one or more predictor variables. It extends linear regression by applying the logistic function to keep predicted probabilities between 0 and 1. Logistic regression estimates the odds that a specific input belongs to a certain class and is valued for its simplicity, interpretability, and ease of use. It assumes a linear relationship between the log odds of the outcome and the predictor variables. The model’s coefficients offer insights into how the independent variables relate to the dependent variable, making it useful for understanding the influence of each feature. Logistic regression can also be used for multi-class classification problems using one-vs-rest or SoftMax regression method. Also, L1 (lasso) and L2 (ridge) regularization techniques are used to prevent overfitting.

#### Decision Tree

Decision Tree (DT)^53^ is a model that splits the data into subsets based on the value of input features, creating a tree-like structure of decisions. Each internal node represents a test on an attribute, each branch represents the outcome of the test, and each leaf node represents a class label or continuous value (regression). DT is easy to interpret and visualize, making it helpful in understanding data structures and predicting outcomes. They can handle both numerical and categorical data and require less preprocessing. However, DT is prone to overfitting, especially with complex trees that capture noise in the training data.

#### Random Forest (RF)

Random Forest (RF)^54^ is an ensemble learning method that constructs multiple decision trees during training and outputs the class selected by majority voting for classification tasks, or the mean prediction for regression tasks. Each tree is trained on a random subset of the training data with replacement (bootstrap sampling), and at each split in the tree, a random subset of features is considered. This randomness helps improve the model’s generalization by reducing variance and avoiding overfitting. RF is robust, easy to use, and often achieves high accuracy. They also provide estimates of feature importance, which can help understand the impact of different features on the predictions. However, they can be computationally expensive and memory-intensive due to the large number of trees and the need to store them.

#### Naive Bayes (NB)

The Naïve Bayes (NB) classifier is a family of probabilistic classification methods based on Bayes’ theorem, incorporating the “naïve” assumption of conditional independence among features given the class label. Despite this strong independence assumption, NB classifiers often perform better in practice, particularly for text classification and spam filtering. They are highly efficient, requiring a linear number of parameters relative to the number of features and classes, and they can be trained quickly, even on small datasets. NB models compute the posterior probability of each class and assign the class with the highest likelihood to the given input^55^.

#### Support Vector Machines (SVM)

Support Vector Machine (SVM) is a supervised learning model used for classification and regression tasks^56^. The goal of SVM is to find the optimal hyperplane that best separates the data into different classes while maximizing the margin between them. Support Vector Machine (SVM) identifies a decision boundary (hyperplane) that maximizes the margin between classes, with the data points closest to the hyperplane, known as support vectors, playing a central role in defining its orientation. For high-dimensional feature spaces, it is well-suited and performs effectively when the number of features exceeds the number of samples. For non-linear classification tasks, kernel functions (e.g., polynomial or radial basis function kernels) are employed to project input data into higher-dimensional spaces where a linear separating boundary can be identified. Despite its flexibility, SVM can be computationally demanding and typically requires careful selection and tuning of kernel and regularization parameters.

#### Artificial Neural Networks (ANN)

Artificial Neural Network (ANN)^57^ computations on models inspired by the neural networks in the human brain. They consist of layers of nodes (neurons), where each connection between nodes has an associated weight. Data is fed into the input layer, processed through hidden layers, and results are produced at the output layer. The learning process involves adjusting the weight using backpropagation algorithms, which reduce errors by updating weights. Artificial Neural Networks (ANNs) are well-suited for modeling complex, non-linear relationships and have been widely applied in domains such as image recognition, speech processing, and natural language understanding. However, ANNs typically require substantial amounts of data and computational resources and may be prone to overfitting if not properly regularized. To address task-specific challenges, various network architectures have been developed, including Convolutional Neural Networks (CNNs) for image-based data and Recurrent Neural Networks (RNNs) for sequential data.

#### K-Nearest Neighbors (KNN)

The K-Nearest Neighbors (KNN) algorithm is a great method used for both classification and regression tasks^58,59^. In classification, KNN assigns a class to an input by considering the majority class among its ‘k’ nearest neighbors in the feature space. For regression, it predicts the value by averaging the closest neighbors. The choice of ‘k’ is crucial because it affects the model’s performance. If ‘k’ is too small, the model may become too sensitive to noise. If ‘k’ is too large, the model might become overly smooth. Finding the right balance is crucial. KNN is popular because it is simple yet effective, especially for certain types of data. However, it can be computationally demanding for large datasets as it requires calculating distances to all training samples. One key advantage of KNN is that it shows improved performance even with noise, complex, and non-linear decision boundaries.

## Model evaluation

The performance of the trained models is evaluated on the testing dataset. All models were assessed under multiple experimental setups to examine the impact of different preprocessing and modeling strategies. The evaluation process includes three experimental setups.

- Data leakage prevention: The dataset was first split into training (80%) and testing (20%) subsets. All preprocessing steps, including SMOTE class balancing and all feature selection methods (ANOVA, Chi-Square, Regression), were performed on the training dataset. The trained models were then evaluated on the untouched testing dataset. No information/data from the testing set was used during the resampling or feature selection process.
- Balanced Dataset: To address class imbalance, the Synthetic Minority Oversampling Technique (SMOTE) was applied to the training set before model training.
- Feature Selection: To improve the model performance, feature selection was performed on the training dataset using ANOVA, Chi-Square, and Regression Analysis. Various combinations of these feature selection methods, including ANOVA, Chi-Square, and Regression Analysis, are also explored. Based on the characteristics of the dataset, experiments are conducted using the following combinations:
  - {ANOVA}, {Chi-Square}, {Regression analysis}
  - {ANOVA ∪ Chi-Square} or (Union of (ANOVA and Chi-Square)).
  - {ANOVA ∪ Regression} or (Union of (ANOVA and Regression)).
  - {Chi-Square ∪ Regression} or (Union of (Chi-Square and Regression)).
  - {ANOVA ∪ Chi-Square ∪ Regression} or (Union of (ANOVA, Chi-Square and Regression)).
  - {ANOVA ∩ Chi-Square} or (Intersection of (ANOVA and Chi-Square)).
  - {ANOVA ∩ Regression} or (Intersection of ANOVA and Regression).
  - {Chi-Square ∩ Regression} or (Intersection of (Chi-Square and Regression)).
  - {ANOVA ∩ Chi-Square ∩ Regression} or (Intersection of (ANOVA, Chi-Square and Regression)).
- Ensemble Techniques: Finally, bagging-based ensemble methods are applied during model training to reduce variance, and the resulting ensemble models are evaluated on the unchanged testing dataset.

To validate the performance of the developed models, the evaluation is conducted in three stages: (i) Performance metrics analysis (accuracy, precision, recall, and F1 score calculated from the confusion matrix), (ii) ROC-AUC analysis, and (iii) Precision comparison. These methods provide a comprehensive understanding of model performance from different perspectives, where the confusion matrix offers detailed insights into model performance by presenting the counts of:

- True Positives (TP): Correctly predicted positive instances.
- True Negatives (TN): Correctly predicted negative instances.
- False Positives (FP): Incorrectly predicted positive instances.
- False Negatives (FN): Incorrectly predicted negative instances.

Based on these values, the following performance metrics are derived:

- Accuracy: Measures the overall correctness of the model’s predictions, calculated through Eq. 1:

1 $$\:Accuracy=\frac{TP+TN}{TP+FN+TN+FP}$$

- Precision: Evaluates the accuracy of positive predictions, calculated through Eq. 2:

2 $$\:Precision=\frac{TP}{TP+FP}$$

- Recall (Sensitivity): Measures the model’s ability to identify all relevant positive instances, calculated through Eq. 3:

3 $$\:Recall=\frac{TP}{TP+FN}$$

- F1 Score: The harmonic means of precision and recall, providing a balanced metric for imbalanced datasets, calculated through Eq. 4.​.

4 $$\:F1\:score=\frac{2*Precision*Recall}{Precision+Recall}$$

Additionally, the Receiver Operating Characteristic (ROC) Curve assesses the diagnostic performance of the models by plotting the true positive rate (recall) against the false positive rate at various thresholds. The Area Under the Curve (AUC) offers a single numerical value that summarizes the model’s ability to differentiate between classes. A higher AUC value indicates better discriminatory ability between classes. The results are shown in tables and graphs for clarity, followed by a detailed discussion.

### Impact of balanced dataset

**Fig. 8:**
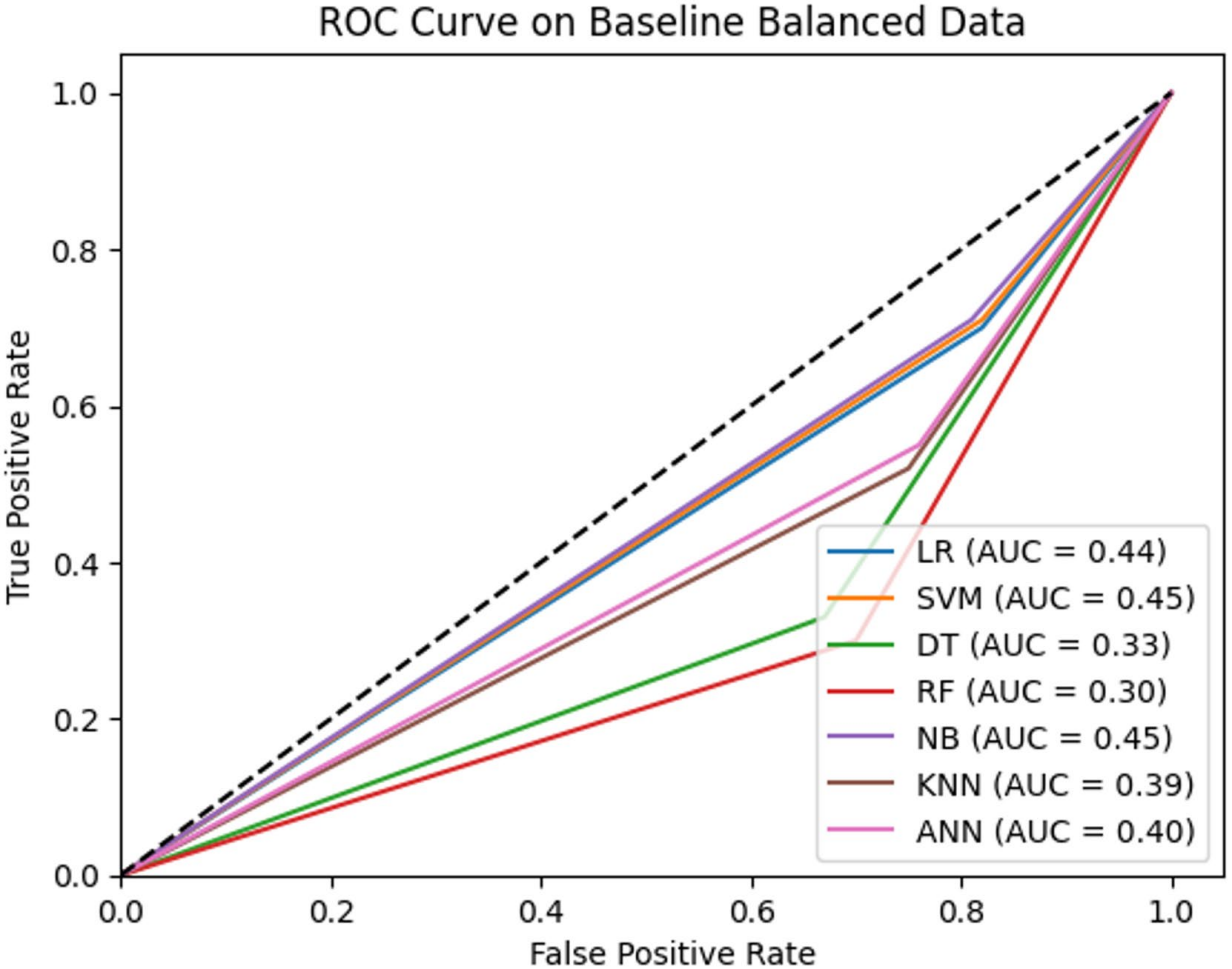
ROC Curves for all models without feature selection and ensemble.

**Fig. 9:**
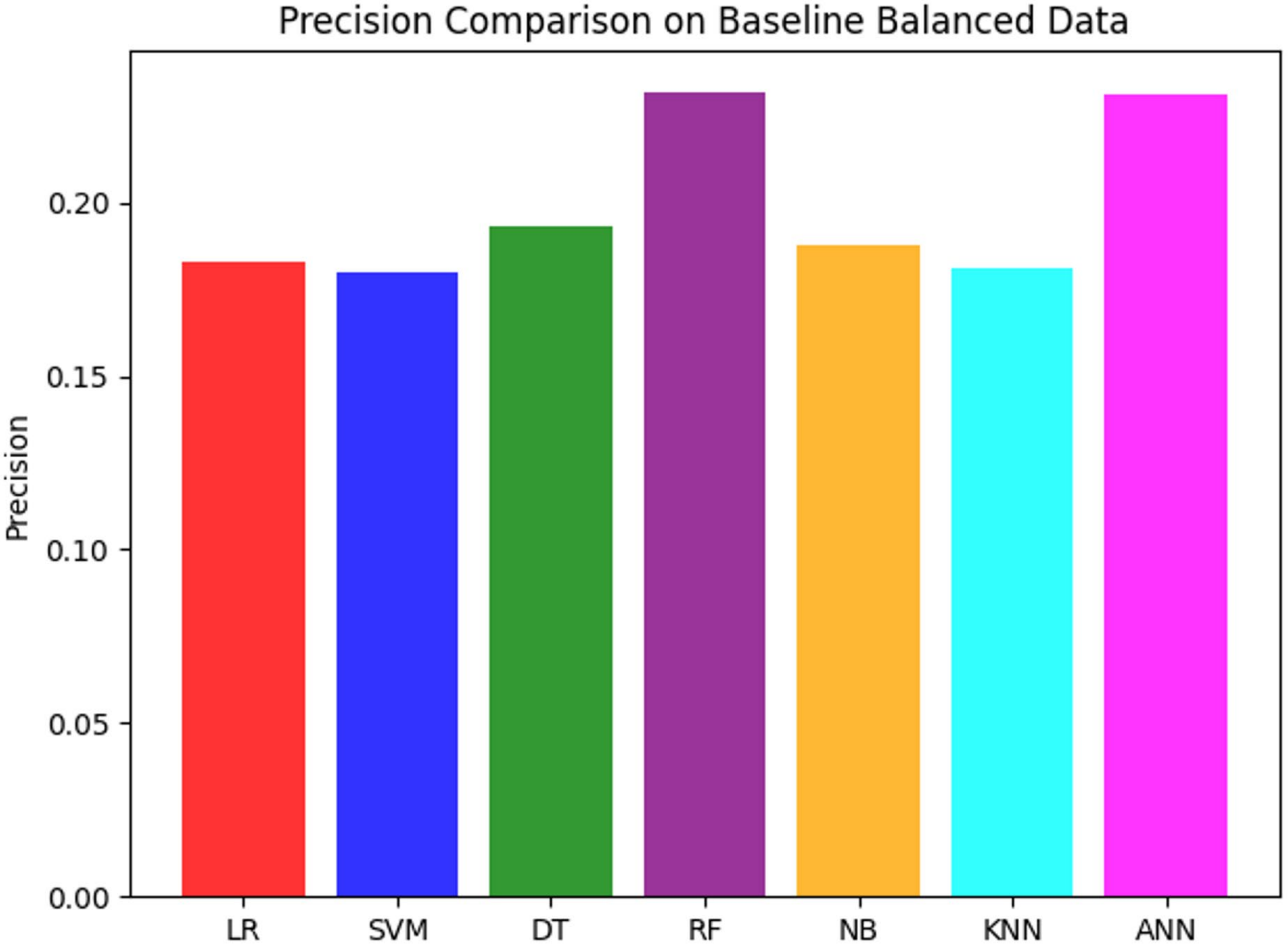
Precision Comparison across models without feature selection and ensemble.

The initial evaluation of various models on a balanced dataset reveals important insights into their performance across accuracy, precision, recall, and F1 score. Table 2 summarizes these performance metrics, while the ROC curve and precision comparison are illustrated in Figs. 8 and 9, respectively.

**Table 2:** Classifier Performance on Balanced Dataset (without feature selection and ensemble).

Models	Accuracy	Precision	Recall	F1 Score
Logistic Regression (LR)	0.701	0.183	0.697	0.289
Decision Trees (DT)	0.821	0.193	0.331	0.243
Random Forest (RF)	0.851	0.232	0.303	0.263
Naive Bayes (NB)	0.709	0.188	0.705	0.297
Support Vector Machine (SVM)	0.694	0.180	0.707	0.288
Artificial Neural Network (ANN)	0.800	0.231	0.548	0.325
K-Nearest Neighbor (KNN)	0.752	0.181	0.522	0.269

Regarding the concept of performance metric analysis, Table 2 shows that Random Forest (RF) has the highest accuracy of 85%, followed by Decision Tree (DT) and Artificial Neural Network (ANN), with accuracy rates of 82% and 80%, respectively. In terms of precision, RF also achieved the highest precision (23%) among the evaluated models, indicating relatively fewer false positives than other classifiers; although, overall precision remains low. However, its recall is relatively low (30%), and it misses many true positives. The F1 score, which balances precision and recall, is highest for ANN (33%), reflecting its better trade-off between these metrics.

Regarding the ROC curve analysis, Fig. 8 further confirms the findings of the performance metrics analysis, where Naïve Bayes (NB) and Support Vector Machine (SVM) achieved the highest AUC scores (45%), underscoring their better overall performance in distinguishing between classes. NB and SVM achieved the highest AUC scores (0.45) among the evaluated models in this setup; however, because these AUC values are below 0.5, the models do not demonstrate meaningful class discrimination under this configuration. Besides, LR and ANN show comparatively better performance than some classifiers in this setup; although, overall AUC values below 0.5 indicate limited discriminatory ability.

Furthermore, Fig. 9 shows the comparison of precision across models. Among the evaluated models, Random Forest (RF) and Artificial Neural Network (ANN) achieved the highest precision values (≈ 23%). Although this suggests relatively fewer false positives than the other models, the absolute precision remains low; therefore, these results do not support claims of reliability. Decision Tree (DT) follows a precision of 19%, while Logistic Regression (LR), Support Vector Machine (SVM), Naïve Bayes (NB), and K-Nearest Neighbor (KNN) exhibit similar precision values around 18%. In summary, RF and ANN achieved the highest precision among the evaluated models; however, low absolute precision indicates that further optimization is required before considering, where minimizing false positives is critical.

Moreover, these analysis results indicate that dataset balancing improves accuracy and recall for several models; however, it alone does not lead to strong discriminatory performance. Although RF and ANN achieve comparatively higher accuracy and precision than other models, absolute precision values remain low, and AUC scores below 0.5 across all classifiers indicate limited class discrimination. These findings suggest that class balancing alone is insufficient for reliable heart disease classification. It highlights the need for additional techniques, such as feature selection or ensemble methods, to further improve model performance.

### Impact of feature selection techniques

This section analyzes the impacts of various feature selection techniques on classifier performance, using performance metrics, ROC curves with AUC scores, and a precision comparison graph. Regarding the matric analysis, Tables 3, 4 and 5 presents the classifiers’ performance in terms of Accuracy, Precision, Recall, and F1 score computed from the confusion matrix of various models across different feature selection and feature combination techniques. The analysis result is demonstrated in detail as follows:

The initial analysis results were obtained by examining three different feature selection techniques: {ANOVA, Chi-Square, and Regression Analysis} (as shown in Table 3). This result indicates that, under the **{ANOVA}** feature selection technique with five ML models, the Random Forest (RF) achieved the highest values across the reported metrics. The K-Nearest Neighbor (KNN) model achieved the highest observed metric, with sufficiently high recall (96%) and F1 score (91%). It suggests that although its precision (87%) is slightly lower than RF and ANN, KNN shows improved performance in identifying positive cases. Decision Tree (DT) shows good accuracy of 89% and performs consistently well across all metrics, but it remains slightly below RF and KNN. Artificial Neural Network (ANN) also shows comparatively balanced performance, with relatively higher precision (88%) and F1 score (87%) under this configuration. Naive Bayes (NB) showed reduced accuracy (77%), precision (75%), recall (80%), and F1 score (77%), indicating lower performance than other models. Logistic Regression (LR) and Support Vector Machine (SVM) also showed comparatively lower performance, making them less suitable for complex classification.

Considering the **{Chi-Square}** feature selection technique, Random Forest (RF) shows higher relative performance (84%) across all four metrics (accuracy, precision, recall, and F1 score), indicating comparatively better performance than the other models. Decision Tree (DT) performs slightly lower than RF but relatively higher in precision and F1 score than other models, indicating that even without ensemble techniques, DT shows improved performance on the selected data. K-Nearest Neighbor (KNN) also shows good performance with a high recall (85%) and an F1 score of 81%; although its precision is slightly lower, its overall performance remains competitive across metrics. Artificial Neural Network (ANN) offers a decent balance across all metrics. Support Vector Machine (SVM) performs similarly to ANN, but slightly lower than other metrics. Naive Bayes (NB) shows useful performance, especially in recall (89%), but struggles with precision (59%). It suggests that while it detects more positive cases with high recall, it also produces more false positives with poor precision. Logistic Regression (LR) underperforms across all metrics compared to the other models.

Considering the **{Regression analysis}** feature selection technique, Random Forest (RF) shows good performance with high accuracy and an F1 score of around 87%, indicating a comparatively better balance between precision and recall. Decision Tree (DT) performs slightly lower than RF but shows relatively consistent performance across all metrics. Its simplicity and interpretability make it a valuable alternative to RF. K-Nearest Neighbor (KNN) shows the highest recall (89%), indicating comparatively higher performance across all metrics, especially in accuracy (85%) and F1 score (86%). Artificial Neural Network (ANN) shows balanced performance with solid metrics, though not as high as RF and DT. However, it has a strong recall (82%) and ability to identify more positive cases, outperforming other models such as LR and NB. The Support Vector Machine (SVM) shows moderate performance across all metrics. It suggests that it is comparatively less effective than ANN, DT, or RF. Logistic Regression (LR) performs nearly identically to SVM. Naive Bayes (NB) exhibits the lowest overall performance, particularly in precision (72%) and recall (78%), indicating its ability to classify many positive cases but overall weak performance.

**Table 3:** Classifier Performance with the selected three feature selection techniques.

Models	Accuracy	Precision	Recall	F1 Score
ANOVA				
Logistic Regression (LR)	0.7660	0.7562	0.7852	0.7704
Decision Tree (DT)	0.8976	0.8888	0.9088	0.8987
Random Forest (RF)	0.9285	0.9237	0.9341	0.9289
Naive Bayes (NB)	0.7709	0.7525	0.8074	0.7790
Support Vector Machine (SVM)	0.7654	0.7544	0.7872	0.7704
Artificial Neural Network (ANN)	0.8730	0.8853	0.8571	0.8710
K-Nearest Neighbor (KNN)	0.9137	0.8726	0.9688	0.9182
Chi-Square				
Logistic Regression (LR)	0.6863	0.6645	0.7522	0.7057
Decision Tree (DT)	0.8344	0.8385	0.8285	0.8334
Random Forest (RF)	0.8410	0.8410	0.8409	0.8410
Naive Bayes (NB)	0.6387	0.5913	0.8980	0.7131
Support Vector Machine (SVM)	0.6865	0.6614	0.7644	0.7092
Artificial Neural Network (ANN)	0.7117	0.6954	0.7533	0.7232
K-Nearest Neighbor (KNN)	0.8073	0.7807	0.8545	0.8160
Regression Analysis				
Logistic Regression (LR)	0.7630	0.7546	0.7796	0.7669
Decision Tree (DT)	0.8638	0.8670	0.8595	0.8632
Random Forest (RF)	0.8720	0.8675	0.8780	0.8727
Naive Bayes (NB)	0.7458	0.7272	0.7868	0.7558
Support Vector Machine (SVM)	0.7630	0.7527	0.7834	0.7677
Artificial Neural Network (ANN)	0.7947	0.7768	0.8270	0.8011
K-Nearest Neighbor (KNN)	0.8574	0.8302	0.8985	0.8630

Following the individual feature selection techniques, we represented the results of different combinations of the selected three feature selection techniques (considering their union and intersection variance). Table 4 presents the results considering the variance of the union of feature selection techniques, following four distinct combinations: {ANOVA ∪ Chi-Square}, {ANOVA ∪ Regression}, {Chi-Square ∪ Regression}, and {ANOVA ∪ Chi-Square ∪ Regression analysis}.

As shown in Table 4, Random Forest (RF) demonstrates the highest accuracy (92%) and F1 score (92%) for the **{ANOVA ∪ Chi-Square}** combination. It shows comparatively better overall performance among the evaluated models. Also, K-Nearest Neighbor (KNN) consistently achieves high scores across all metrics for this combination, with strong accuracy (91%), precision (87%), and recall (96%). However, this result is nearly equivalent to the RF score, indicating comparable performance under this configuration. Decision Tree (DT) also shows improved performance, achieving a moderate accuracy of 89%, precision of 88%, and recall of 90%. Artificial Neural Network (ANN) performs competitively, with comparatively higher accuracy (87%) than SVM and LR. Logistic Regression (LR) and Support Vector Machine (SVM) show identical accuracy of 76%. However, RF achieves the highest precision (92%) and recall (93%), demonstrating a higher F1 score and accuracy.

For the **{ANOVA ∪ Regression}** combination, Random Forest (RF) shows improved performance across all metrics with higher accuracy (89%), precision (87%), and recall (91%). Although it suggests more true positives, it also indicates a large number of false positives. The Decision Tree (DT) performed better with strong accuracy (87%) and a well-balanced recall score, which is slightly lower than RF. Artificial Neural Networks (ANN) achieve an average accuracy of 82%, indicating balanced performance with minimal deviation. K-Nearest Neighbor (KNN) achieves the best F1 score (94%) among all models, showing its consistent performance. Although it has a comparatively higher F1 score and average precision (83%), it has relatively low recall (74%), which suggests variability. Naive Bayes (NB) shows moderate accuracy (77%) with good recall (80%). Support Vector Machine (SVM) and Logistic Regression (LR) perform similarly, with average accuracy around 76%. SVM and LR perform lower than tree-based models across all metrics under these settings.

For the **{Chi-Square ∪ Regression} **combination, Random Forest (RF) shows comparatively higher overall accuracy (91%), precision (89%), recall (92%), and F1 score (91%). Decision Tree (DT) also performs strongly with 85% accuracy and a higher recall (89%). It correctly identifies a large number of positives. However, its precision is slightly lower than RF, but it maintains a solid F1 score (86%), indicating good overall performance. K-Nearest Neighbor (KNN) shows a balanced performance (high accuracy (81%), recall (91%), and F1 score (83%)), though its precision is lower (76%). Naive Bayes (NB) shows good recall but a moderate reduction in precision. Artificial Neural Network (ANN) provides balanced results across all metrics; however, it is not a higher classification model. Logistic Regression (LR) and Support Vector Machine (SVM) have nearly identical average accuracy (76%), reflecting their interpretability, but neither is the most accurate model.

For the **{ANOVA ∪ Chi-Square ∪ Regression}** combination, Random Forest (RF) achieved the highest scores across all evaluation metrics (accuracy of 92%, precision of 92%, and F1 Score of 92%). However, it is slightly lower in recall at 93%, compared to K-Nearest Neighbor (KNN). Decision Tree (DT) achieved the highest observed metric, with high precision (84%) and an F1 score of 87%, indicating a strong ability to identify positive cases. KNN boasts a very high recall (94%) but a slightly lower F1 score (83%), followed by DT. It also has somewhat lower accuracy (81%) and precision (74%). Artificial Neural Network (ANN) is characterized as a balanced and consistent model with an accuracy of 82%, precision of 83%, recall of 81%, and F1 Score of 82%. Naive Bayes (NB) shows moderate accuracy, precision, recall, and F1 score, reflecting its adequacy but not a strong model like RF or DT. Support Vector Machine (SVM) and Logistic Regression (LR) perform similarly, with lower performance across all metrics (accuracy of 76%, precision of 75%, recall of 78%, and F1 Score of 77%).

**Table 4:** Classifier Performance with the union of the selected three feature selection techniques.

Models	Accuracy	Precision	Recall	F1 Score
ANOVA ∪ Chi-Square				
Logistic Regression (LR)	0.7660	0.7562	0.7852	0.7704
Decision Tree (DT)	0.8979	0.8892	0.9091	0.8990
Random Forest (RF)	0.9285	0.9241	0.9338	0.9289
Naive Bayes (NB)	0.7710	0.7526	0.8074	0.7790
Support Vector Machine (SVM)	0.7654	0.7544	0.7872	0.7704
Artificial Neural Network (ANN)	0.8725	0.8633	0.8852	0.8741
K-Nearest Neighbor (KNN)	0.9137	0.8726	0.9688	0.9182
ANOVA ∪ Regression Analysis				
Logistic Regression (LR)	0.7660	0.7562	0.7852	0.7704
Decision Tree (DT)	0.8747	0.8488	0.9119	0.8792
Random Forest (RF)	0.8949	0.8777	0.9178	0.8973
Naive Bayes (NB)	0.7710	0.7526	0.8074	0.7790
Support Vector Machine (SVM)	0.7654	0.7544	0.7872	0.7704
Artificial Neural Network (ANN)	0.8224	0.8300	0.8109	0.8203
K-Nearest Neighbor (KNN)	0.8113	0.8342	0.7438	0.9497
Chi-Square ∪ Regression Analysis				
Logistic Regression (LR)	0.7646	0.7556	0.7821	0.7687
Decision Tree (DT)	0.8564	0.8297	0.8968	0.8620
Random Forest (RF)	0.9113	0.8997	0.9259	0.9126
Naive Bayes (NB)	0.7498	0.7120	0.8389	0.7702
Support Vector Machine (SVM)	0.7646	0.7542	0.7850	0.7693
Artificial Neural Network (ANN)	0.7874	0.7833	0.7947	0.7890
K-Nearest Neighbor (KNN)	0.8186	0.7668	0.9158	0.8347
ANOVA ∪ Chi-Square ∪ Regression Analysis				
Logistic Regression (LR)	0.7660	0.7562	0.7852	0.7704
Decision Tree (DT)	0.8747	0.8488	0.9119	0.8792
Random Forest (RF)	0.9285	0.9241	0.9338	0.9289
Naive Bayes (NB)	0.7710	0.7526	0.8074	0.7790
Support Vector Machine (SVM)	0.7654	0.7544	0.7872	0.7704
Artificial Neural Networks (ANN)	0.8224	0.8300	0.8109	0.8203
K-Nearest Neighbor (KNN)	0.8113	0.7438	0.9497	0.8342

Additionally, Table 5 presents the results of the intersection of feature selection techniques across four distinct combinations: {ANOVA ∩ Chi-Square}, {ANOVA ∩ Regression}, {Chi-Square ∩ Regression}, and {ANOVA ∩ Chi-Square ∩ Regression analysis}.

As shown in Table 5, this result indicates that for the **{ANOVA ∩ Chi-Square} **combination, Random Forest (RF) demonstrates high overall performance across all metrics (accuracy, precision, recall, and F1 score are around 84%). This indicates that RF achieves higher accuracy and balanced performance under the {ANOVA ∩ Chi-Square} feature selection approach. Decision Tree (DT) also shows improved performance with good accuracy (79%), recall (87%), and F1 score (80%). Although scores are slightly lower than RF, they’re balanced across the data. This suggests that the DT model effectively identifies true positives and minimizes false positives, indicating comparatively higher performance in this context. Artificial Neural Networks (ANN) show relatively better performance, particularly in recall (81%), indicating good sensitivity. It slightly underperforms Random Forest (RF) but still maintains a good balance across all metrics. It’s a good choice for more complex data patterns. K-Nearest Neighbor (KNN) performs closely to ANN in terms of F1 score (72%) and recall (82%), but they’re slightly lower in precision and accuracy. It offers a good trade-off between recall and precision, demonstrating its effectiveness in capturing true positives. Naive Bayes (NB) has the highest recall (94%) among all models. It can capture nearly all true positive cases. However, with a lower precision (63%), it also reflects a higher number of false positives. Logistic Regression (LR) shows moderate performance across all metrics. It has relatively higher recall (75%) than precision, indicating the model is better at identifying positive cases. Support Vector Machine (SVM) exhibits similar performance to LR, with slightly better recall. However, it does not perform as well as tree-based or ensemble models.

For the **{ANOVA ∩ Regression}** combination, Random Forest (RF) shows the highest average performance across all four metrics with accuracy (81%), precision (79%), recall (85%), and F1 score (82%). Beσiδeσ, Naive Bayes (NB) and Decision Tree (DT) show similar performance with high accuracy. These models achieved higher average performance for both generalization and balance between precision and recall. The K-Nearest Neighbor (KNN) performs moderately well, particularly showing significant improvement in recall (83%). Support Vector Machine (SVM) and Logistic Regression (LR) offer decent results but fall short compared to ensemble models and tree-based methods. Artificial Neural Network (ANN) shows underfitting with the lowest scores across all metrics.

For the **{Chi-Square ∩ Regression}** combination, Random Forest (RF) and Decision Tree (DT) have the highest overall balanced performance across all four metrics (accuracy 69%, precision 67%, recall 77%, and F1 score 72%). Both models performed identically and showed comparatively better performance in this classification task. Naive Bayes (NB) achieved the highest recall (94%), which outperformed other models. However, its low precision (58%) indicates that it misses many positives but also generates many false positives. Artificial Neural Network (ANN) showed good performance, especially in F1 score (70%), indicating a balanced trade-off between precision and recall. Logistic Regression (LR) and Support Vector Machine (SVM) perform almost identically across all metrics, indicating consistent performances but not outstanding. The K-Nearest Neighbor (KNN) shows the lowest score across most metrics, particularly in recall (67%) and F1 score (65%). It appears less effective on this dataset compared to others.

For the **{ANOVA ∩ Chi-Square ∩ Regression} **combination, Random Forest (RF) and Decision Tree (DT) achieve the highest accuracy (69%) and F1 scores (72%), which indicates comparatively balanced performance across evaluation metrics. Beσiδeσ, Naive Bayes (NB) achieves the highest recall (94%) and F1-score (72%), but the lowest precision (58%), which indicates that it detects almost all positives but produces many false positives. Logistic Regression (LR) and Support Vector Machine (SVM) have nearly identical performance, with moderate balance across all metrics. Also, Artificial Neural Network (ANN) shows comparatively better precision and F1 score than LR and SVM. K-Nearest Neighbor (KNN) showed the lowest overall performance across accuracy (64%), recall (67%), and F1-score (65%).

**Table 5:** Classifier Performance with the intersection of the selected three feature selection techniques.

Models	Accuracy	Precision	Recall	F1 Score
ANOVA ∩ Chi Square				
Logistic Regression (LR)	0.6862	0.6645	0.7522	0.7056
Decision Tree (DT)	0.7903	0.7501	0.8705	0.8058
Random Forest (RF)	0.8410	0.8409	0.8411	0.8410
Naive Bayes (NB)	0.6976	0.6327	0.9419	0.7569
Support Vector Machine (SVM)	0.6865	0.6614	0.7644	0.7092
Artificial Neural Networks (ANN)	0.7522	0.7228	0.8181	0.7675
K-Nearest Neighbor (KNN)	0.7432	0.7083	0.8267	0.7630
ANOVA ∩ Regression Analysis				
Logistic Regression (LR)	0.7630	0.7546	0.7796	0.7669
Decision Tree (DT)	0.8147	0.7987	0.8414	0.8195
Random Forest (RF)	0.8169	0.7962	0.8519	0.8231
Naive Bayes (NB)	0.8147	0.7987	0.8414	0.8195
Support Vector Machine (SVM)	0.7630	0.7527	0.7834	0.7677
Artificial Neural Network (ANN)	0.7561	0.7343	0.8026	0.7669
K-Nearest Neighbor (KNN)	0.7850	0.7602	0.8327	0.7948
Chi-Square ∩ Regression Analysis				
Logistic Regression (LR)	0.6615	0.6442	0.7213	0.6806
Decision Tree (DT)	0.6977	0.6701	0.7787	0.7204
Random Forest (RF)	0.6978	0.6699	0.780	0.7208
Naive Bayes (NB)	0.6364	0.5846	0.9427	0.7217
Support Vector Machine (SVM)	0.6612	0.6430	0.7252	0.6816
Artificial Neural Network (ANN)	0.6831	0.6626	0.7462	0.7019
K-Nearest Neighbor (KNN)	0.6494	0.6430	0.6716	0.6570
ANOVA ∩ Chi-Square ∩ Regression Analysis				
Logistic Regression (LR)	0.6615	0.6442	0.7213	0.6806
Decision Tree (DT)	0.6977	0.6701	0.7787	0.7204
Random Forest (RF)	0.6978	0.6699	0.7800	0.7208
Naive Bayes (NB)	0.6364	0.5846	0.9427	0.7217
Support Vector Machine (SVM)	0.6612	0.6430	0.7252	0.6816
Artificial Neural Network (ANN)	0.6831	0.6626	0.7462	0.7019
K-Nearest Neighbor (KNN)	0.6494	0.6430	0.6716	0.6570

**Fig. 10:**
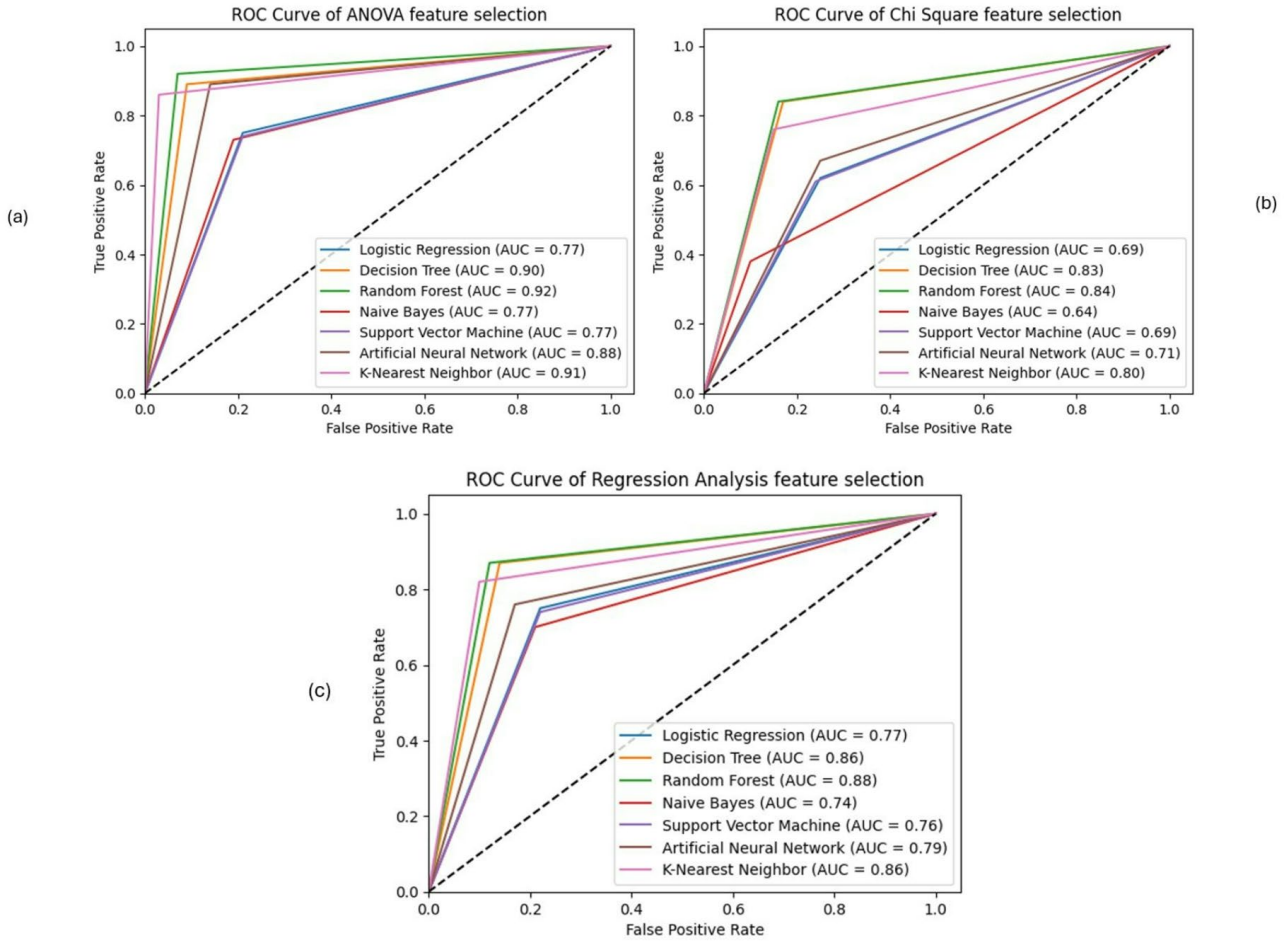
ROC Curves analysis for seven distinct models with three feature selection techniques, where figure (**a**) reflects (ANOVA); figure (b) reflects (Chi-Square) combination; and figure (**c**) reflects (Regression analysis).

Regarding the ROC curve analysis, Fig. 10 shows the ROC curves and AUC scores for the classifiers across four different feature selection methods. It indicates that each model achieved its highest AUC with the ANOVA feature selection method. Specifically, Random Forest (RF) and Decision Tree (DT) achieved higher AUC scores (0.92 and 0.90), indicating improved class discrimination under this configuration. This shows that in the tested dataset, ANOVA-based feature selection yields improved class discrimination compared to Chi-Square and Regression-based feature selection analysis for several models. Artificial Neural Network (ANN) also shows improved performance with ANOVA, with an AUC score of 0.88, indicating slightly lower performance than with the Chi-Square feature selection. However, Naive Bayes (NB) performance was comparatively poorer than others, with an AUC score of 0.77. Overall, Chi-Square results tend to be lower, especially for Logistic Regression (LR) (0.69), Naïve Bayes (NB) (0.64), and ANN (0.71). Conversely, Regression analysis performs better than Chi-Square but worse than ANOVA, with good accuracy for LR (0.77) and Support Vector Machine (0.76).

**Fig. 11:**
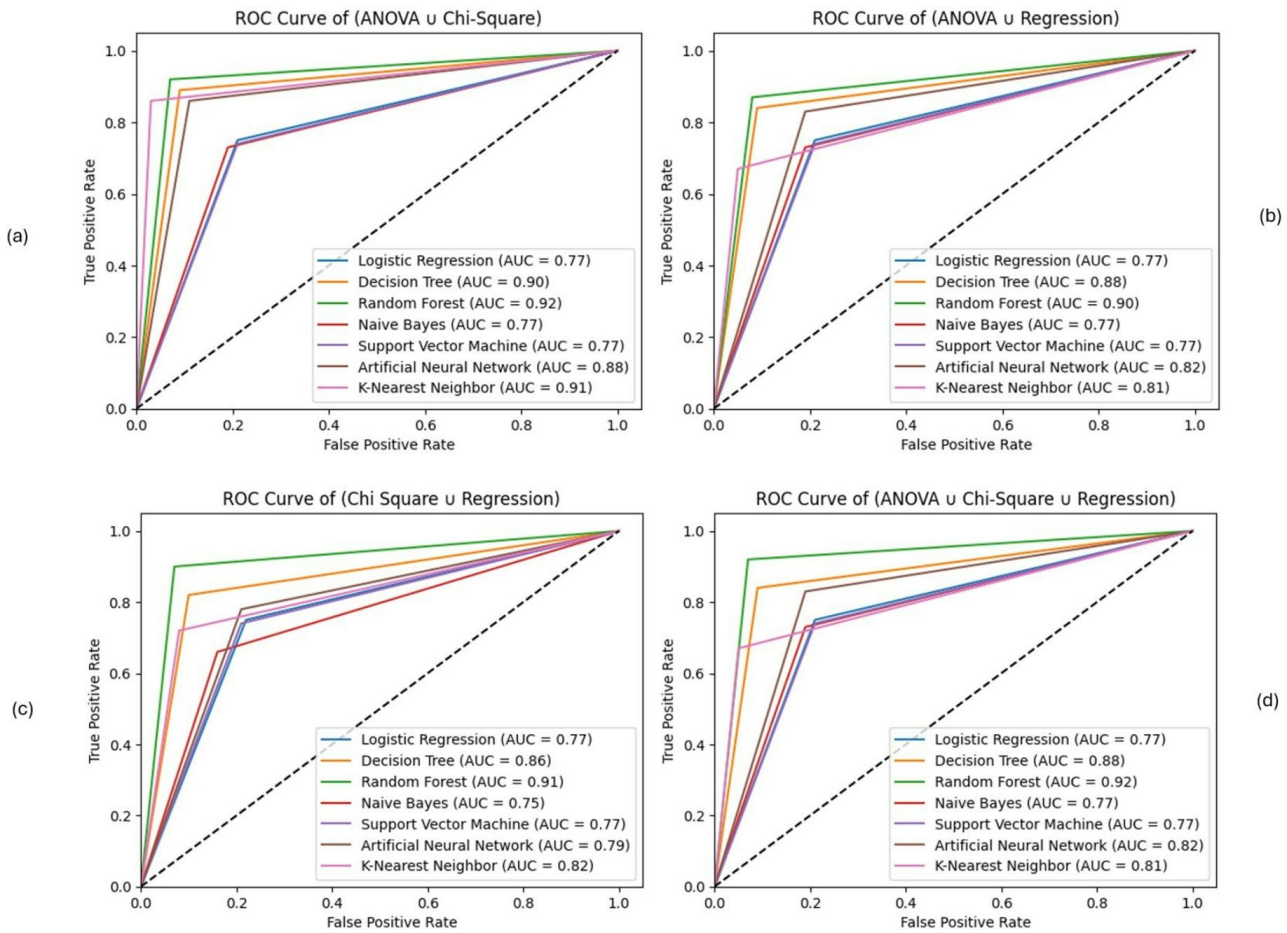
ROC Curves analysis for seven distinct models with different combinations of feature selection techniques, where figure (**a**) reflects (ANOVA ∪ Chi-Square) combination; figure (**b**) reflects (ANOVA ∪ Regression) combination; figure (**c**) reflects (Chi-Square ∪ Regression) combination, and figure (**d**) reflects (ANOVA ∪ Chi-Square ∪ Regression) combination.

Figure 11 illustrates the ROC curves and AUC scores for the classifiers across four distinct combinations, particularly the union of feature selection techniques. This figure shows that Random Forest (RF) consistently achieves the highest AUC scores across all feature selection combinations. Notably, the (ANOVA ∪ Chi-Square) and (ANOVA ∪ Chi-Square ∪ Regression) feature selection combination resulted in the highest AUC score (0.92). Decision Tree (DT) also shows higher AUC scores for (ANOVA ∪ Chi-Square) feature selection, with AUC scores of 0.90. It indicates improved class discrimination. However, Logistic Regression (LR), Naïve Bayes (NB), and Support Vector Machine (SVM) showed performance variability. Besides, Artificial Neural Network (ANN) and K-Nearest Neighbor (KNN) showed comparatively better performance in the combination of (ANOVA ∪ Chi-Square) feature selection with AUC scores of 0.88 and 0.91, respectively.

**Fig. 12:**
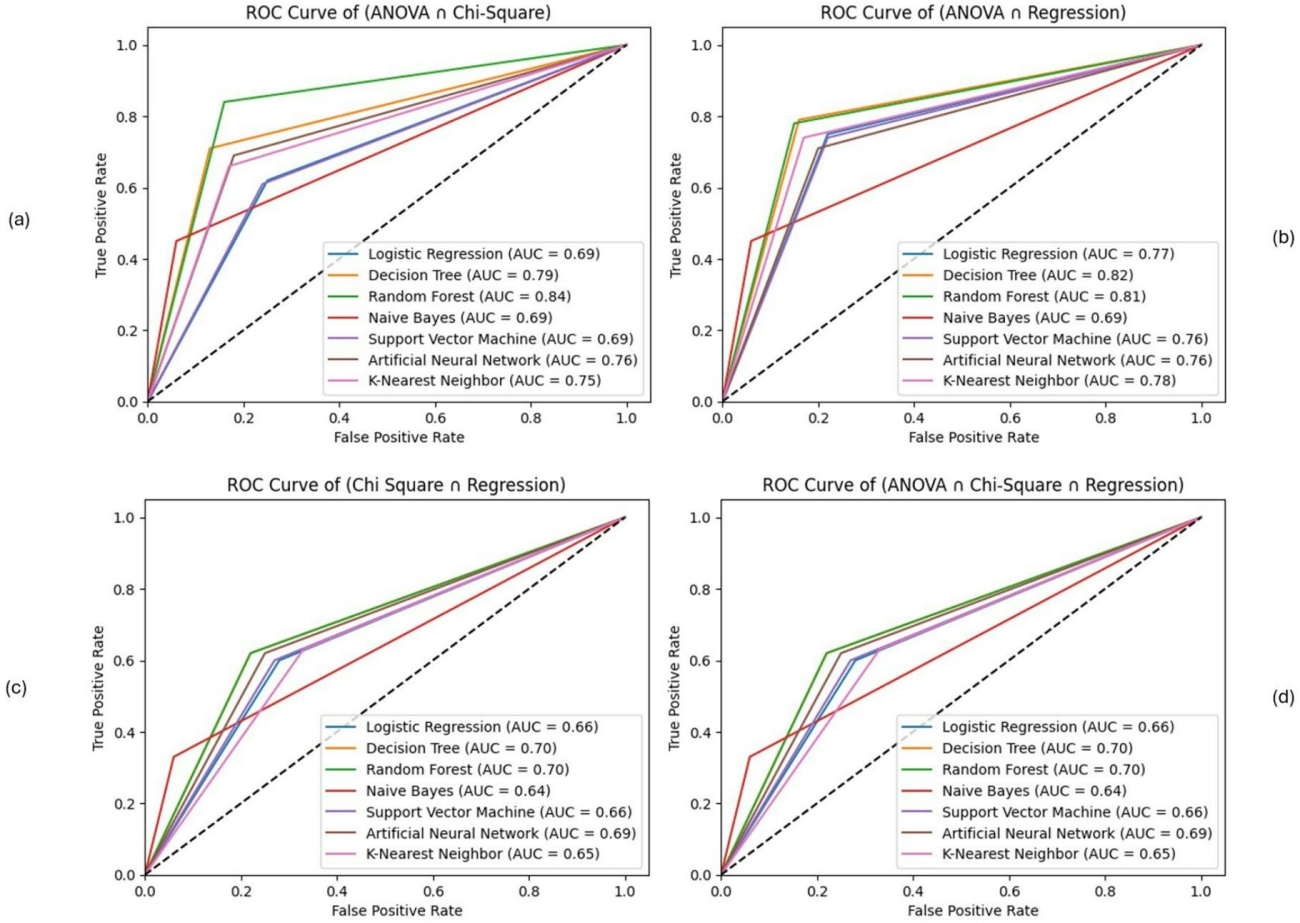
ROC Curves analysis for seven distinct models with different combinations of feature selection techniques, where figure (**a**) reflects (ANOVA ∩ Chi-Square) combination; figure (**b**) reflects (ANOVA ∩ Regression) combination; figure (**c**) reflects (Chi-Square ∩ Regression) combination, and figure (**d**) reflects (ANOVA ∩ Chi-Square ∩ Regression) combination.

Figure 12 shows that the AUC scores of all seven classifiers are higher for the combination of (ANOVA ∩ Chi-Square) feature selection techniques, with Random Forest (RF) achieving the highest AUC score of 0.84. Additionally, Decision Tree (DT) exhibits a good AUC score of 0.82 for the (ANOVA ∩ Regression) feature selection combination. However, Logistic Regression (LR), Decision Tree (DT), Support Vector Machine (SVM), and K-Nearest Neighbor (KNN) achieved higher AUC scores of 0.77, 0.82, 0.76, and 0.78, respectively, for the same (ANOVA ∩ Regression) feature selection combination. All models consistently show lower AUC scores when considering (Chi-Square ∩ Regression) and (ANOVA ∩ Chi-Square ∩ Regression) feature selection techniques.

**Fig. 13:**
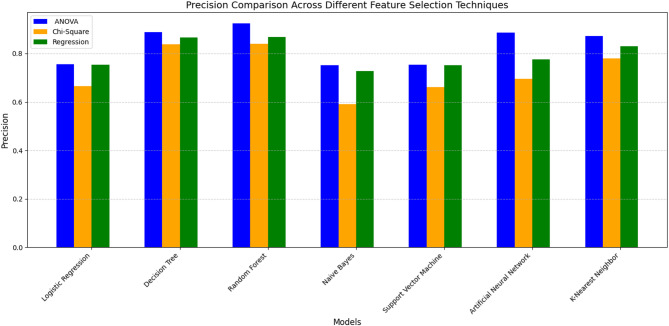
Precision Comparison across models with three feature selection techniques.

Addressing the comparison of precision values, Fig. 13 compares the precision of classifiers across three feature selection techniques. Figure 13 shows that {ANOVA} yielded the highest precision among the evaluated feature selection methods for the Random Forest (RF) algorithm with an accuracy of around 92%. Additionally, most models showed good precision for this particular feature selection method. In contrast, {Chi-Square} shows comparatively lower performance, especially for Naïve Bayes (NB), Logistic Regression (LR), and Support Vector Machine (SVM). {Regression analysis} shows moderate performance with better precision than Chi-Square but lower than ANOVA, indicating it retains useful features but may miss some important ones or patterns.

**Fig. 14:**
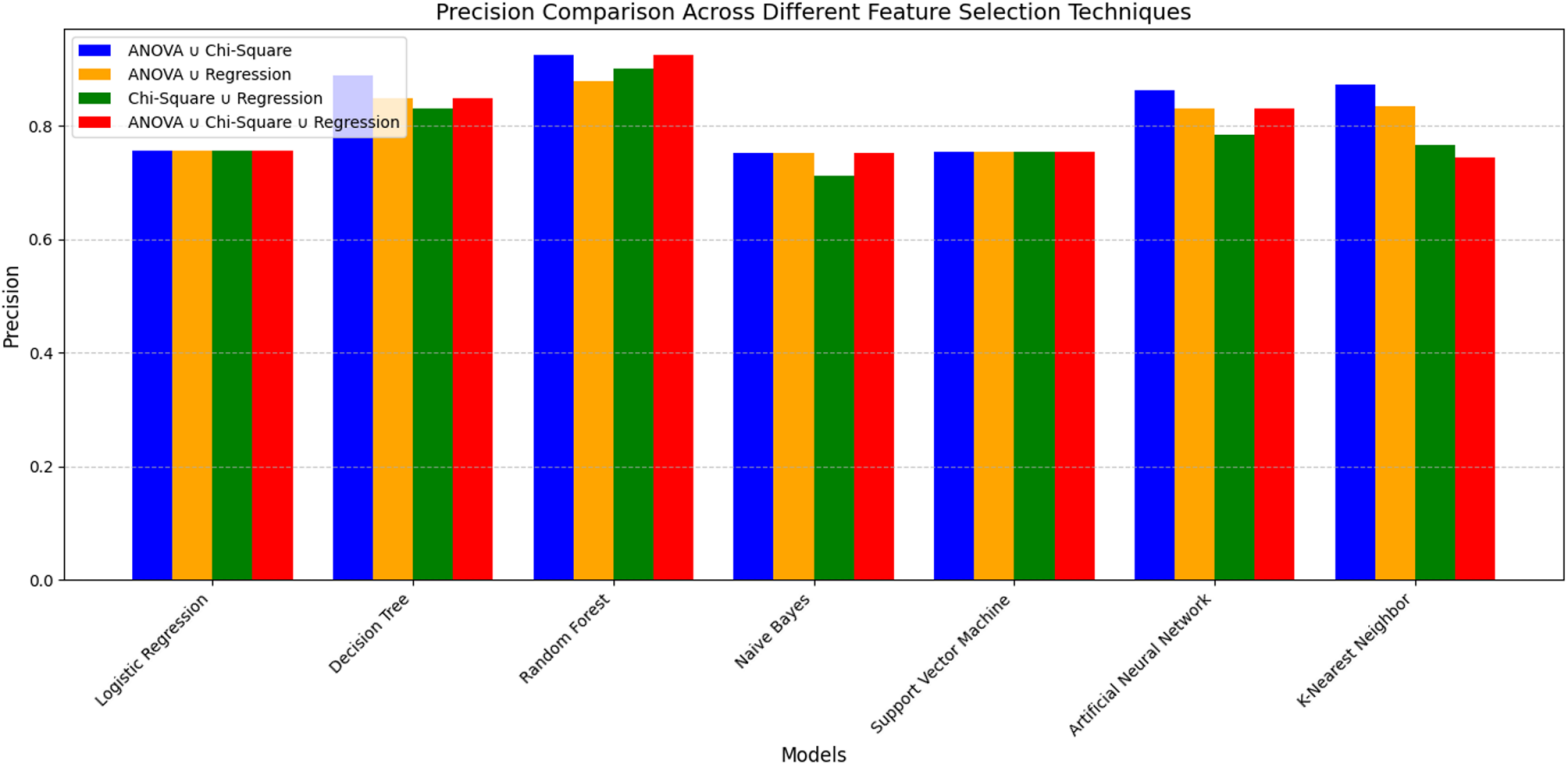
Precision Comparison across models with three feature selection techniques (variance of union).

Figure 14 shows comparative precision results for seven classifiers across different feature combinations, highlighting the variation in feature unions derived from three feature selection techniques. It depicts that Random Forest (RF) consistently achieves high precision across all the evaluated feature-selection combinations. It achieves the highest precision (92%), particularly for the combination of (ANOVA ∪ Chi Square) and (ANOVA ∪ Chi Square ∪ Regression) feature selection techniques. It also shows consistently higher performance for the (ANOVA ∪ Regression) and (Chi Square ∪ Regression) combination with precisions of 87% and 89%, respectively. This indicates that RF achieves higher precision across multiple feature-selection combinations. Decision Tree (DT) also shows higher precision (88%) for the combination of (ANOVA ∪ Chi-Square), although it is slightly lower in other combinations. However, for the combination of (ANOVA ∪ Chi Square), Artificial Neural Network (ANN) exhibits a slightly lower precision (86%) compared to RF and DT. Logistic Regression (LR) and Naïve Bayes (NB) also demonstrate stable but lower performance across all models. Moreover, RF shows the largest observed increase in precision, peaking in the most complex feature combination. Other models generally exhibit moderate precision and sometimes perform poorly with complex feature combinations, indicating lower accuracy and precision. Overall, the highest precision scores are observed for (ANOVA ∪ Chi-Square) and (ANOVA ∪ Chi-Square ∪ Regression) combination, leading to better classifier precision in most cases.

**Fig. 15:**
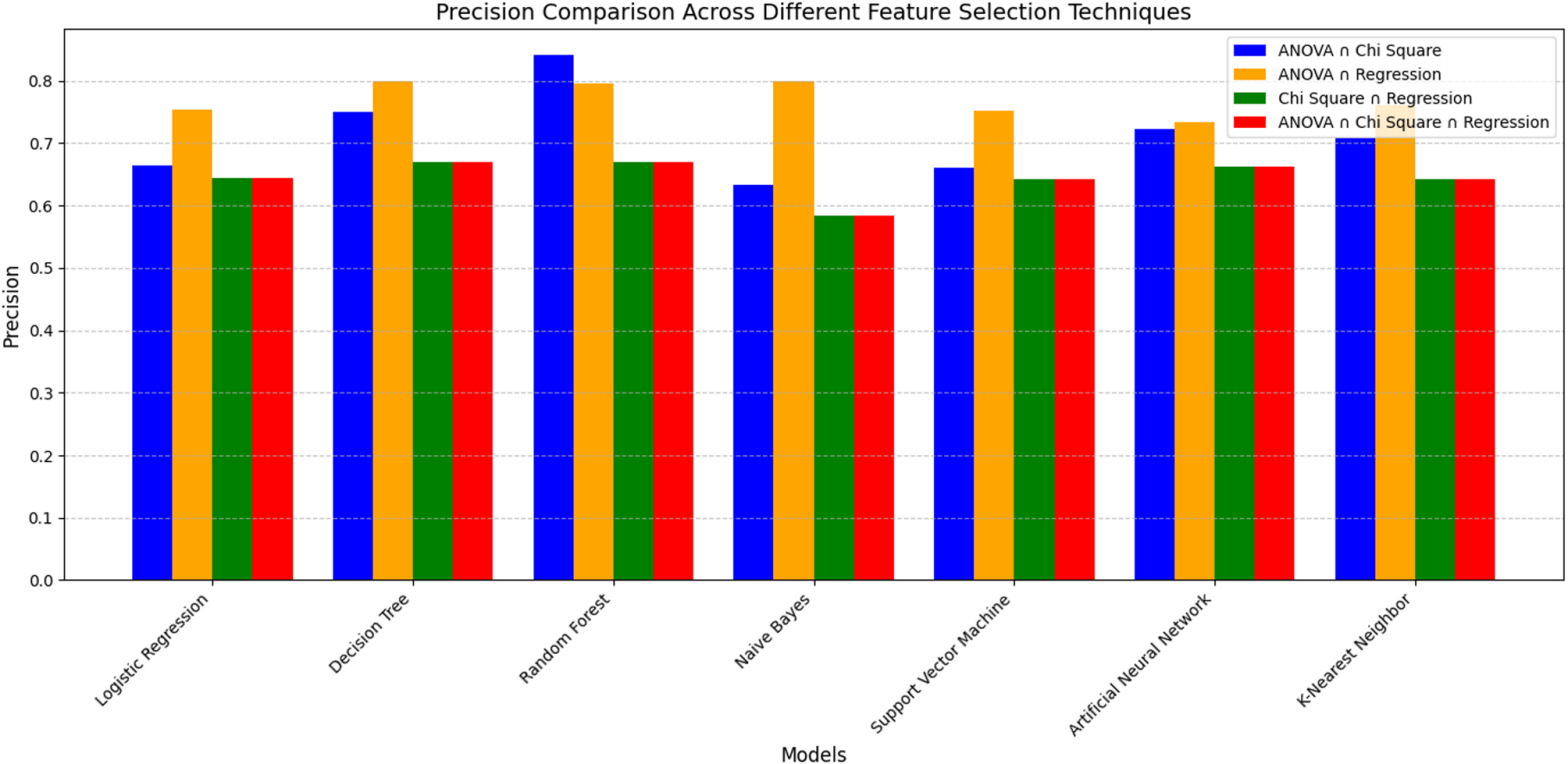
Precision Comparison across models with three feature selection techniques (variance of intersection).

Figure 15 shows that for the {ANOVA ∩ Chi-Square} feature selection technique, Random Forest (RF) achieves the highest precision (84%). Besides, all the other models perform moderately. For the {ANOVA ∩ Regression analysis} feature selection technique, Naïve Bayes (NB) and Decision Tree (DT) achieve the highest performance with 79% precision. In this case, most models showed balanced precision. This suggests that combining {ANOVA ∩ Regression analysis} helps retain features associated with higher precision values. For the {Chi-Square ∩ Regression analysis} feature selection techniques, all models show the lowest performance, with precision below 67%. This indicates that this feature selection combination lacks variance and resulted in comparatively lower performance. Interestingly, the {ANOVA ∩ Chi-Square ∩ Regression analysis} feature selection technique yields performance similar to that of {Chi-Square ∩ Regression analysis}.

Moreover, this analysis suggests that appropriate feature selection techniques influence classifier performance and can affect precision, recall, and AUC scores. The {ANOVA} feature selection method with (ANOVA ∪ Chi-Square), and (ANOVA ∪ Chi-Square ∪ Regression) feature selection combination consistently yields more balanced performance across evaluation metrics, with Random Forest (RF) achieving comparatively higher results across multiple configurations. Decision Tree (DT), Naive Bayes (NB), and Logistic Regression (LR) also showed improved performance in specific scenarios. It highlights the importance of selecting appropriate feature selection methods that align with the dataset and goals.

### Impact of ensemble technique with bagging

**Fig. 16:**
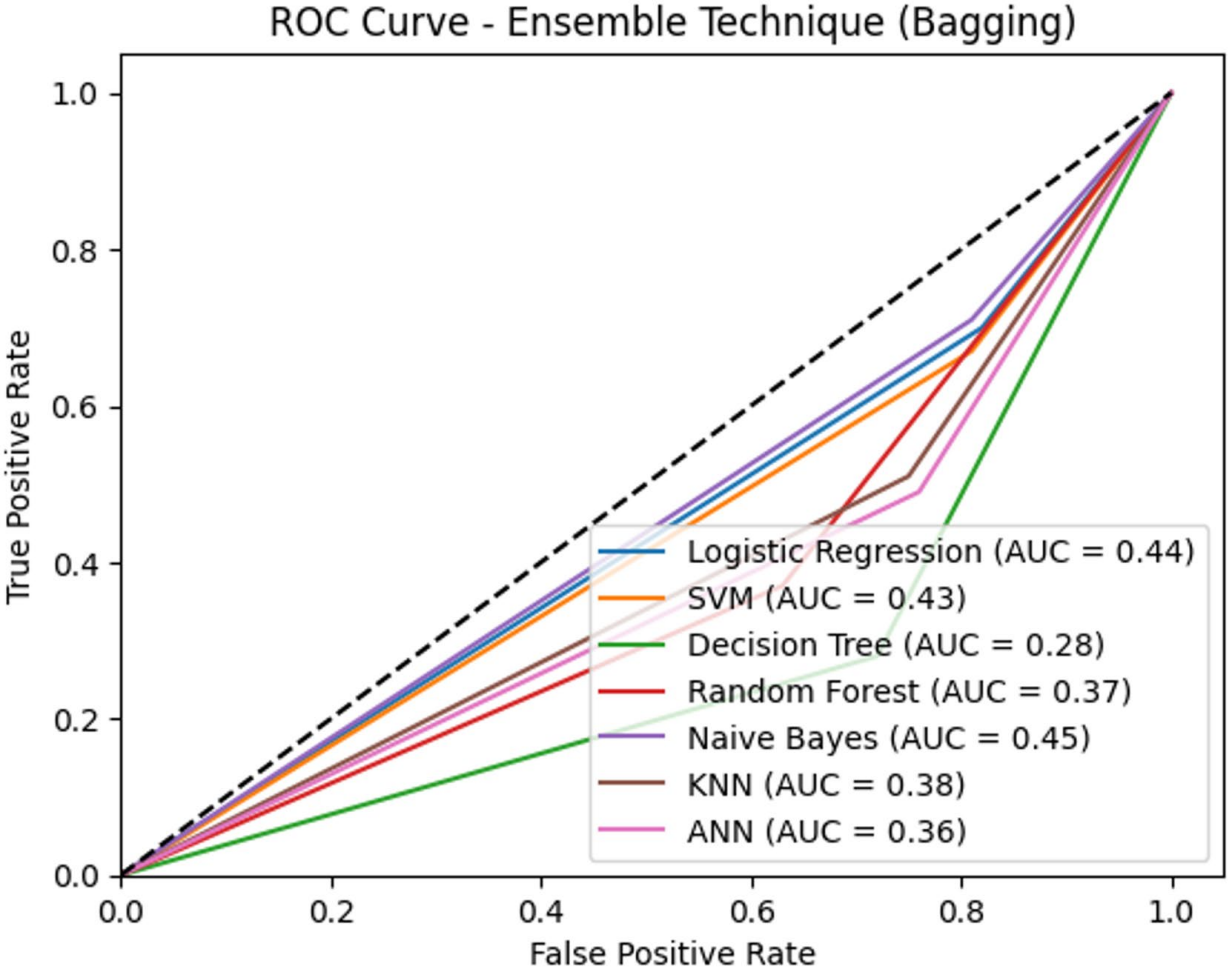
ROC Curves for all models while considering the ensemble.

**Fig. 17:**
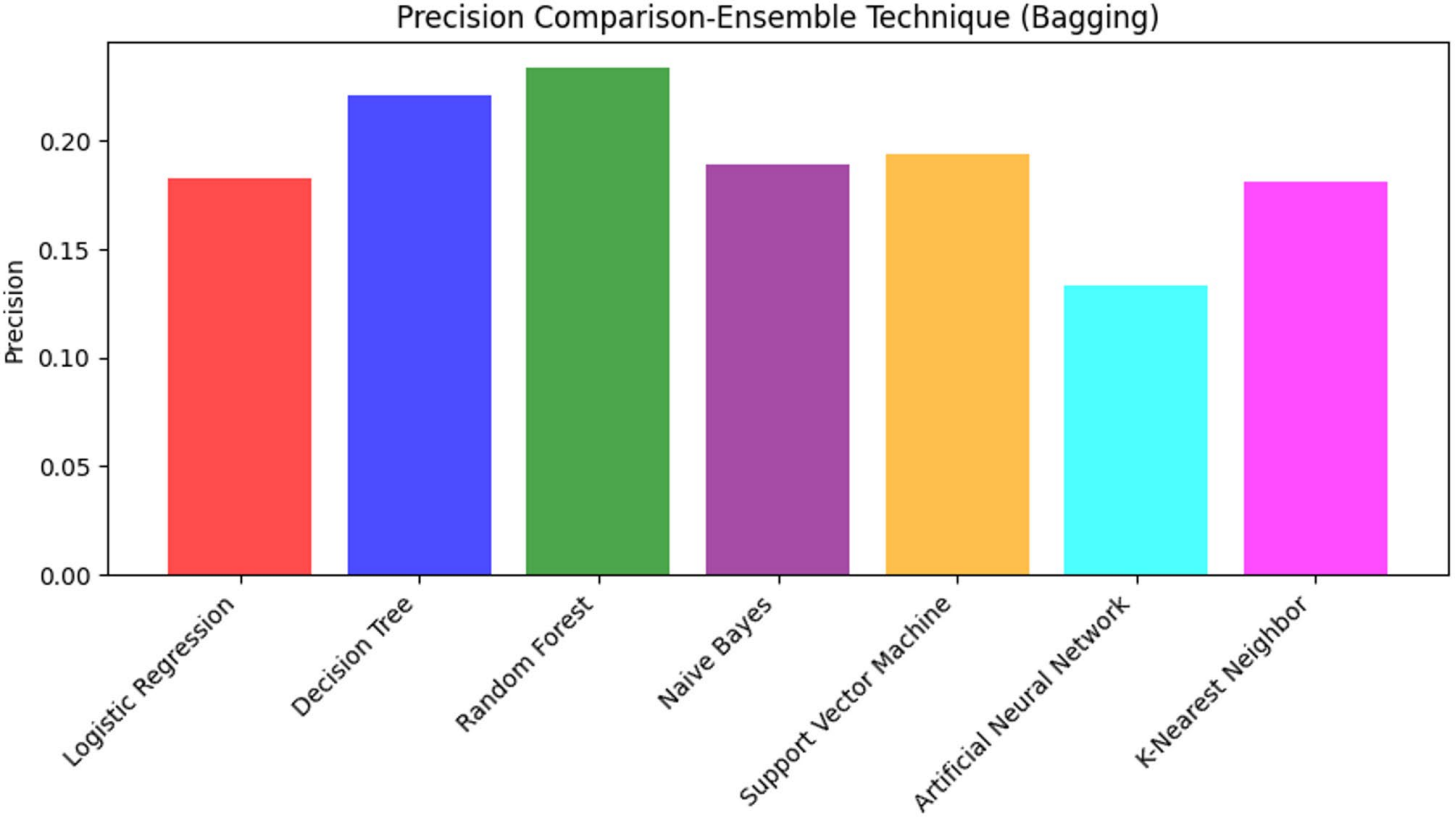
Precision Comparison across models with the ensemble technique.

The application of the ensemble technique using bagging showed mixed impacts on model performance, as summarized in Table 6. Besides, Figs. 16 and 17 provide further insights through ROC-AUC analysis and precision comparison, respectively.

Addressing the performance matrices, Table 6 presents the performance analysis of various models with the bagging ensemble technique. Among the models, Decision Tree (DT) achieves the highest accuracy, approximately 85%, followed by Random Forest (RF) and Artificial Neural Network (ANN) with accuracy of around 84% and 79%, respectively. In terms of precision, RF achieved the highest score (23%), indicating its ability to detect fewer false positives than other models, although the overall precision remains low. However, its relatively lower recall (37%) suggests it misses a considerable number of true positives. The F1 Score, which balances precision and recall, is higher for SVM at approximately 30%, demonstrating its ability to maintain a balanced precision and recall.

**Table 6:** Classifier performance with ensemble technique (bagging).

Model	Accuracy	Precision	Recall	F1 Score
Logistic Regression (LR)	0.701	0.183	0.697	0.289
Decision Tree (DT)	0.851	0.221	0.276	0.245
Random Forest (RF)	0.839	0.234	0.368	0.288
Naive Bayes (NB)	0.709	0.189	0.706	0.298
Support Vector Machine (SVM)	0.728	0.194	0.672	0.301
Artificial Neural Network (ANN)	0.793	0.133	0.488	0.256
K-Nearest Neighbor (KNN)	0.754	0.181	0.514	0.268

The ROC curve analysis (as shown in Fig. 16) also corroborates these findings, highlighting that Naïve Bayes (NB) achieved the highest AUC score (0.45). However, since AUC is below 0.5, this configuration does not demonstrate meaningful performance. Following NB, Logistic Regression (LR) and Support Vector Machine (SVM) show comparatively higher AUC values than other models, since AUC values below 0.5 indicate limited predictive usefulness. However, none of the classifiers showed significant performance, as the AUC score is lower than 0.5 across all metrics, indicating non-significant performance.

Additionally, Fig. 17 illustrates the precision comparison of models under the bagging ensemble technique. Random Forest (RF) again leads with the highest precision (23%), followed closely by Decision Tree (DT), which is around 22%. Support Vector Machine (SVM) and Logistic Regression (LR) show moderate performance with precision values of approximately 19% and 18%, respectively. Naïve Bayes (NB) and K-nearest Neighbor (KNN) showed similar precision scores (18%), while Artificial Neural Network (ANN) demonstrates the lowest precision, around 13%. This analysis indicates that RF achieves relatively fewer false positives than other models, although overall precision remains low.

Overall, the ensemble technique with bagging demonstrates a mixed and limited impact on model performance. Although bagging improves accuracy for certain models, particularly DT and RF, it does not substantially enhance precision or class discrimination. Across all classifiers, precision remains low, and AUC values below 0.5 indicate limited or non-informative discriminatory capability. These results suggest that, when used alone, bagging is insufficient to produce reliable predictive performance for this dataset and should be combined with additional strategies, such as feature selection, to achieve more meaningful improvements.

### Discussion

In this study, we evaluated the performance of several machine learning (ML) algorithms for earlier heart disease classification. The ‘BRFSS 2020 Heart Disease Dataset’ was utilized for model training and testing. Specifically, we employed seven widely used ML algorithms, including Logistic Regression (LR), Decision Tree (DT), Random Forest (RF), Naïve Bayes (NB), Support Vector Machine (SVM), Artificial Neural Network (ANN), and K-Nearest Neighbor (KNN) to assess their performance under different experimental configurations, including class balancing, feature selection, and ensemble learning. We considered three feature selection methods, including ANOVA, Chi-Square, and Regression analysis. To assess the performance of the ML algorithms, we used several evaluation metrics, including confusion matrix analysis, ROC curve analysis, and precision comparison.

The experimental results show that, based on the confusion matrix (accuracy, precision, recall, and F1 score), RF performed comparatively better than other models across several experimental setups. It also shows that, with feature selection techniques, particularly ANOVA, RF showed improved recall and achieved the highest accuracy (92%). Additionally, RF achieved a good F1 score, indicating a better balance between precision and recall than other models within the same experimental setup. This suggests that feature selection techniques have a notable impact on model performance. However, they have different outcomes when considering different combination-based feature selection methods. Notably, RF achieved the highest observed accuracy (92%) and comparatively higher precision and recall under the specific feature selection setups, such as (ANOVA), (ANOVA ∪ Chi-Square), and (ANOVA ∪ Chi-Square ∪ Regression). Models, such as DT, KNN, and ANN, also showed notable performance improvements. Specifically, the ANOVA feature selection technique combined with different methods improved model accuracy, particularly with DT, which achieved the highest accuracy of 89%. DT also achieved a higher F1 score, indicating a relatively improved balance between false positives and false negatives.

According to the ROC curve analysis, RF generally achieved higher AUC values than other models in several experimental setups. For the balanced dataset, none of the models performed well, with AUC scores below 0.5, indicating poor classification performance. Additionally, a balanced dataset, combined with feature selection techniques and their various combinations, improved the model’s ability to classify instances appropriately. In contrast, other models showed variability and underperformed in certain setups. Moreover, ensemble techniques with bagging yield comparatively higher AUC values for LR, NB, and SVM than other models; however, all AUC values remain below 0.5, indicating limited discriminatory capability. The ROC curve analysis indicates relative differences in model performance rather than definitive reliability in the classification tasks for this domain. However, none of the classifiers demonstrate discriminatory performance with the initial technique, as all AUC scores are below 0.5, indicating lower performance.

Regarding precision comparisons across different setups, RF is identified as the model with the highest observed precision, making it comparatively suitable for reducing false positives. Using a balanced dataset, models sufficiently improved recall but often showed lower precision, highlighting the need for further optimization through feature selection. Additionally, the balanced dataset with feature selection techniques, especially regression-based methods, such as {ANOVA}, {ANOVA ∪ Chi-Square}, and {ANOVA ∪ Chi-Square ∪ Regression}, resulted in more balanced performance across evaluation metrics. RF achieved its highest accuracy and improved recall under this setup, and DT also improved with these techniques. Ensemble methods provided consistent gains in accuracy and precision, with RF and DT emerging as comparatively stronger performers within the evaluated models. Moreover, for heart disease classification, RF generally achieved higher relative performance across multiple metrics and experimental setups, particularly with feature selection techniques. Ensemble techniques also affect performance metrics, making them valuable in fine-tuning performance. To reduce false positives, LR and DT, when combined with inclusive feature selection, achieved sufficiently higher precision. However, further optimization would be required before practical deployment. Overall, data balancing with feature selection techniques showed higher average metrics and relative performance.

However, to answer the first research question (RQ1), balanced datasets and ensemble techniques performed well, with variation across the models. In contrast, the balanced dataset, considering feature selection techniques and their specific combinations, produced consistent results across the models. Specifically, the {ANOVA} feature selection technique showed a notable improvement over Chi-Square and Regression feature selection methods. Additionally, various feature selection combinations, such as {ANOVA ∪ Chi-Square} and {ANOVA ∪ Chi-Square ∪ Regression}, demonstrate notable improvements in the model performance, confirming that appropriate feature selection can increase prediction accuracy. To address the second research question (RQ2), several ML models showed performance improvements under specific preprocessing and experimental setups. Among these, RF consistently achieved higher metrics, especially when combined with data balancing and feature selection techniques. Moreover, RF also showed higher relative performance across several configurations due to its classification ability. Other models showed different results across scenarios, indicating inconsistent performance. This may be due to the nature of the data and the classification capabilities of these models with such datasets. The key findings of this comparative analysis are shown below:

Regarding the impact of different preprocessing strategies (balanced datasets, feature selection, and ensemble techniques) [RQ1]:

- Class balancing alone comparatively improves recall but does not provide effective class discrimination, as AUC values remain below 0.5. In contrast, feature selection, particularly ANOVA-based combined approaches, consistently improves accuracy, recall, F1 score, and AUC values across multiple models.
- Feature selection has a greater influence on overall model performance than bagging-based ensemble techniques, which provide mixed benefits and limited improvement in precision and class discrimination when used independently.

Regarding the performance of the selected seven ML models (LR, DT, RF, NB, SVM, ANN, and KNN) [RQ2]:

- Random Forest (RF) achieves comparatively higher relative performance across several metrics and experimental configurations, especially when combined with feature selection. Besides, Decision Tree (DT) and K-Nearest Neighbor (KNN) also show better performance for specific feature selection strategies.
- Persistently low precision values in some configurations and AUC scores below 0.5 indicate that, despite relative improvements, further optimization and validation are required before practical or clinical deployment.

### Practical implications

The findings of this study have important implications for methodological comparison and future research. A comparatively greater performance of feature selection methods over ensemble techniques suggests that focusing on relevant features is more crucial than complex model combinations for heart disease classification. This insight could guide future methodological developments in automated decision-support research.

### Limitations and future work

It is important to acknowledge certain limitations of the study. The study was conducted using the BRFSS 2020 Heart Disease Dataset, and the results may vary when applied to different datasets or populations. Future research could investigate the generalizability of these findings across diverse datasets, including more recent BRFSS releases (e.g., the 2024 dataset), and evaluate the potential of these models in clinical decision support systems. Therefore, in our future work, we aim to investigate hybrid approaches combining the best-performing feature selection methods with optimized ensemble techniques; explore deep learning architectures with the identified optimal feature sets; develop interpretable models that can provide explanations for their predictions, enhancing their utility in clinical settings; and validate these approaches on larger, more diverse datasets to ensure broader applicability.

## Conclusion

This research presents a comparative analysis of machine learning approaches for heart disease classification. Our evaluation of seven machine learning algorithms, combined with various feature selection methods and ensemble techniques, yielded several comparative observations. The experimental results indicate that feature selection techniques, particularly {ANOVA}, {ANOVA ∪ Chi-Square}, and {ANOVA ∪ Chi-Square ∪ Regression}, improved model performance compared to baseline implementations. Under these configurations, Random Forest (RF) achieved the highest observed accuracy (92%) and recall (93%) among the evaluated models, while Decision Tree (DT) showed a comparatively similar accuracy of 89%. These results suggest that appropriate feature selection can improve classification accuracy and may support more stable model behavior. While ensemble techniques based on bagging showed mixed results across models, they provided performance improvements for certain high-variance algorithms, such as the Decision Tree (DT). However, the impact of bagging varied across models, suggesting that its application should be carefully considered across different algorithms and use cases. Notably, some models showed lower precision with bagging, indicating a potential trade-off between performance metrics. In conclusion, this research provides comparative insights into the use of machine learning techniques and preprocessing methods for heart disease classification. The findings may inform future research aimed at improving predictive models, although further validation across diverse datasets is required before considering clinical application.

## Data Availability

The dataset analyzed during the current study is publicly available. The ‘BRFSS 2020 Heart Disease Dataset’ can be accessed through the Zenodo repository at [https://zenodo.org/records/15364962](https:/zenodo.org/records/15364962) . No additional restrictions apply to the use of the data.
